# Triggered Assembly of a DNA-Based Membrane Channel

**DOI:** 10.1021/jacs.1c06598

**Published:** 2022-03-07

**Authors:** Conor Lanphere, Jonah Ciccone, Adam Dorey, Nora Hagleitner-Ertuğrul, Denis Knyazev, Shozeb Haider, Stefan Howorka

**Affiliations:** † Department of Chemistry, Institute of Structural Molecular Biology, University College London, London WC1H 0AJ, United Kingdom; ‡ Institute of Applied Experimental Biophysics, Johannes Kepler University, 4040 Linz, Austria; § Department of Pharmaceutical and Biological Chemistry, University College London School of Pharmacy, London WC1N 1AX, United Kingdom

## Abstract

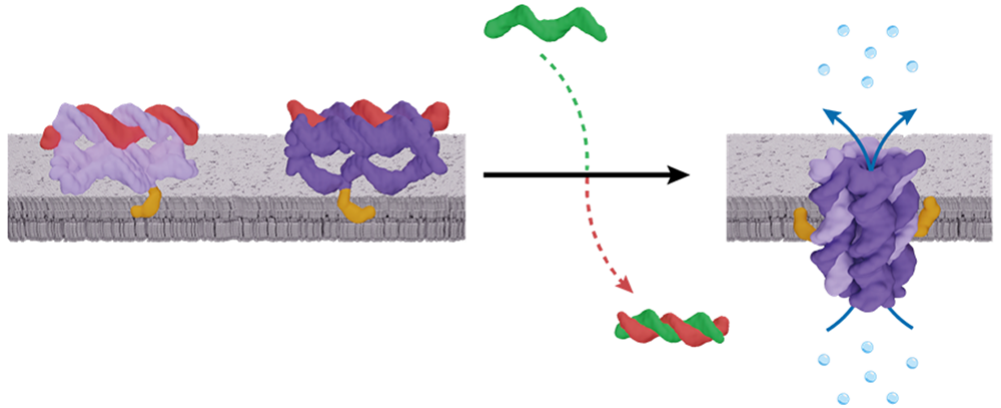

Chemistry is in a powerful position to synthetically replicate biomolecular structures. Adding functional complexity is key to increase the biomimetics’ value for science and technology yet is difficult to achieve with poorly controlled building materials. Here, we use defined DNA blocks to rationally design a triggerable synthetic nanopore that integrates multiple functions of biological membrane proteins. Soluble triggers bind via molecular recognition to the nanopore components changing their structure and membrane position, which controls the assembly into a defined channel for efficient transmembrane cargo transport. Using ensemble, single-molecule, and simulation analysis, our activatable pore provides insight into the kinetics and structural dynamics of DNA assembly at the membrane interface. The triggered channel advances functional DNA nanotechnology and synthetic biology and will guide the design of controlled nanodevices for sensing, cell biological research, and drug delivery.

## Introduction

Replicating complex biological functions via simple and tunable synthetic means is of considerable interest in science and technology. The myriads of biological nanopores^1,2^ and other membrane proteins^3,4^ are a powerful inspiration in this endeavor. By forming a water-filled channel, protein nanopores shuttle bioactive cargo across cell membranes and provide scientific insight into transport and molecular interaction within confined space. In technology, nanopores have helped pioneer portable and scalable DNA sequencing by allowing individual nucleic acids strands to pass a reading head.^5−7^ Nanopores are also used in the sensing of non-DNA analytes.^8−12^ Reflecting these strengths, synthetic pores have been created with self-assembling peptides,^13−16^ organic molecules,^17−20^ or inorganic materials^21^ in order to broaden the sensing range^22,23^ and to understand how transport is influenced by pore chemistries, shapes, and sizes not accessible in biology.

Nanopores are, however, often constitutively open, which limits their functional complexity. To address this constraint, barrel-like pores may be equipped with a lid that can be removed or opened by an external stimulus. As a potential disadvantage, such pores might be leaky for cargo in the nominally closed state or cause leakage when inserted into bilayers and related semifluid membranes. Leakiness can reduce the pores’ application potential in analyte sensing, drug delivery, or targeted cell lysis.

A more powerful way to advance the function is controlling the formation of membrane-spanning pores using an exogenous trigger. The controlled assembly of nonspanning subunits into a barrel-like pore is functionally complex and offers a clear turn-on signal that avoids leakage of lidded pores. The trigger-assembled pores would also add scientific breadth by integrating several fundamental processes that underpin their formation: (i) molecular recognition—between the trigger and the pore subunits to activate them for interaction; (ii) conformational changes—of pore subunits at the membrane to prime them for interaction; and (iii) molecular assembly—of the activated subunits to form a pore membrane-spanning pore that transports cargo. By integrating the three processes, the triggerable synthetic pores would mimic the actions of many dedicated membrane proteins that are specialized to carry out these tasks.

DNA nanotechnology is a tested and versatile route for biomimetic design. DNA nanotechnology offers high structural precision, tunability, and dynamic-nanomechanical control^24−29^ along with chemical modifications for expanding functional interactions with biomolecules,^30^ including bilayers. Building on these strengths, rational designs with DNA have yielded barrel-like membrane pores with tunable lumen diameters.^31−43^ The structural dynamics of DNA nanopores and their molecular interaction with the bilayer membranes has been studied with molecular dynamics simulations^44−48^ complementing other computational studies on biological pores.^49,50^ Designs with DNA have also led to pores that unblock the channel lumen in response to stimuli, such as oligonucleotides,^32^ proteins,^51^ or temperature,^52^ and controllably capped nanotubes.^53^ However, DNA membrane nanopores with a controlled assembly have not been built so far. These DNA structures could be used for sensing, cell biological research, or drug delivery. These *in situ* assembled DNA pores could also aid our understanding of how molecular recognition and assembly of DNA at membrane interfaces alter the kinetics and structural dynamics in the pore’s assembly pathway.

**Figure 1:**
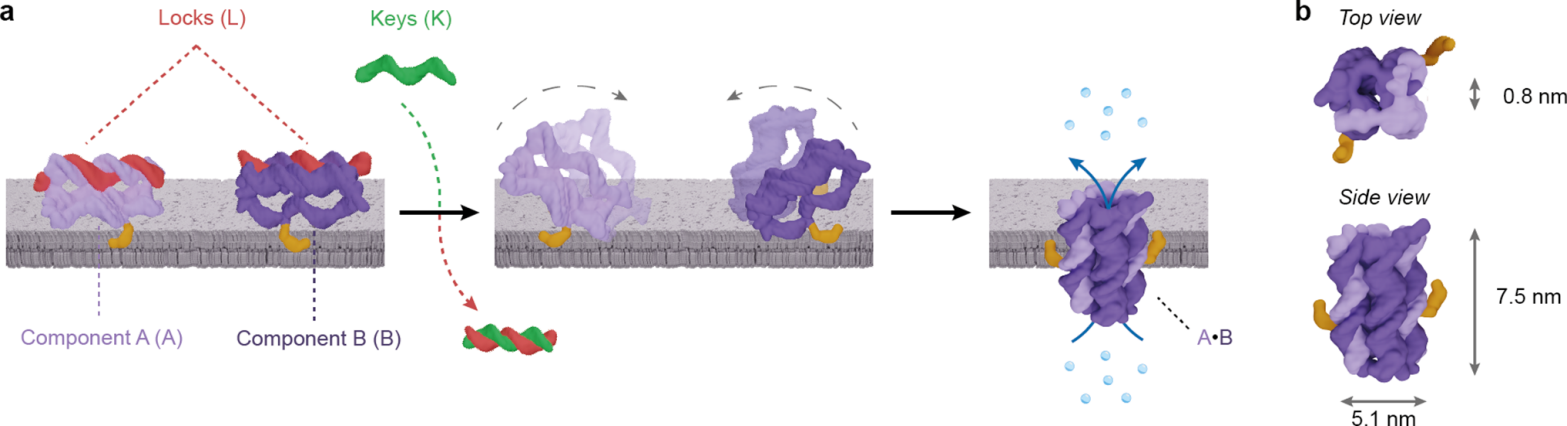
Triggered assembly of DNA nanopore A•B from components A and B at the membrane interface. (a) Assembly-inactive components AL^A^ (light purple, red) and BL^B^ (dark purple, red) carrying locks L^A^ and L^B^ are tethered to the membrane via a cholesterol anchor (orange-yellow). The addition of key strands K^A^ and K^B^ (green) unzips the lock strands to allow activated A and B to reorient relative to the bilayer and form a membrane-spanning ion channel. (b) Top-down and side views of the A•B pore featuring four DNA helices arranged in a square lattice. The membrane cholesterol anchors are in orange-yellow.

Here, we enlist DNA nanotechnology to construct a functionally advanced membrane pore that assembles from two unique subunits after triggered activation (Figure 1). The controlled formation integrates the processes of molecular recognition between the triggers and the inactive subunits, the repositioning of the activated subunits within the membrane, and their assembly into a functional channel (Figure 1). We determine the affinity and kinetics of pore formation to reveal any influence of the lipid bilayer on the pathway. Furthermore, molecular dynamics simulations explore the structural dynamic changes associated with repositioning of the membrane-bound DNA components during assembly. We finally assay fluorophore and ion flux to probe the efficiency of transport across the assembled DNA channel.

## Results and Discussion

### DNA Nanopore Design

We designed a DNA nanopore capable of assembling from constituent components at the membrane interface (Figure 1) and in solution. The nanopore, denoted A•B, consists of a bundle of four DNA helices and assembles from components A and B. Each component is made from two DNA strands that form a central duplex, a ssDNA loop and two ssDNA arms (Figure 1, Figures S1 and S2, Tables S1 and S2). The pore assembles from the components by hybridization between the ssDNA arms of one component and the loop of the other component, thereby forming two additional duplexes. The resulting pore’s nominal dimensions are 7.5 nm in height and 5.1 nm in outer width. The inner channel lumen is up to 0.8 nm wide.

To control pore formation, the components can be rendered assembly-inactive with two lock strands, L^A^ and L^B^. In the inactive components AL^A^ and BL^B^, the lock strands sequester the ssDNA arms in a second duplex (Figure 1, Figure S2), thus rendering them incapable of binding the other component. However, the lock strands feature a 10-nucleotide overhang which allows for their selective removal by addition of a key strand via toehold-mediated-strand displacement (Figure 1, Figure S2).^54^ The addition of key strands, K^A^ and K^B^, restores the ability of components A and B to form a pore (Figure 1). To facilitate membrane binding, each component carries a cholesterol anchor. After pore assembly, the cholesterol anchors are symmetrically positioned on opposite sides of the pore (Figure 1, Figure S2).

### A•B Assembles Directly or via Triggered Activation

**Figure 2:**
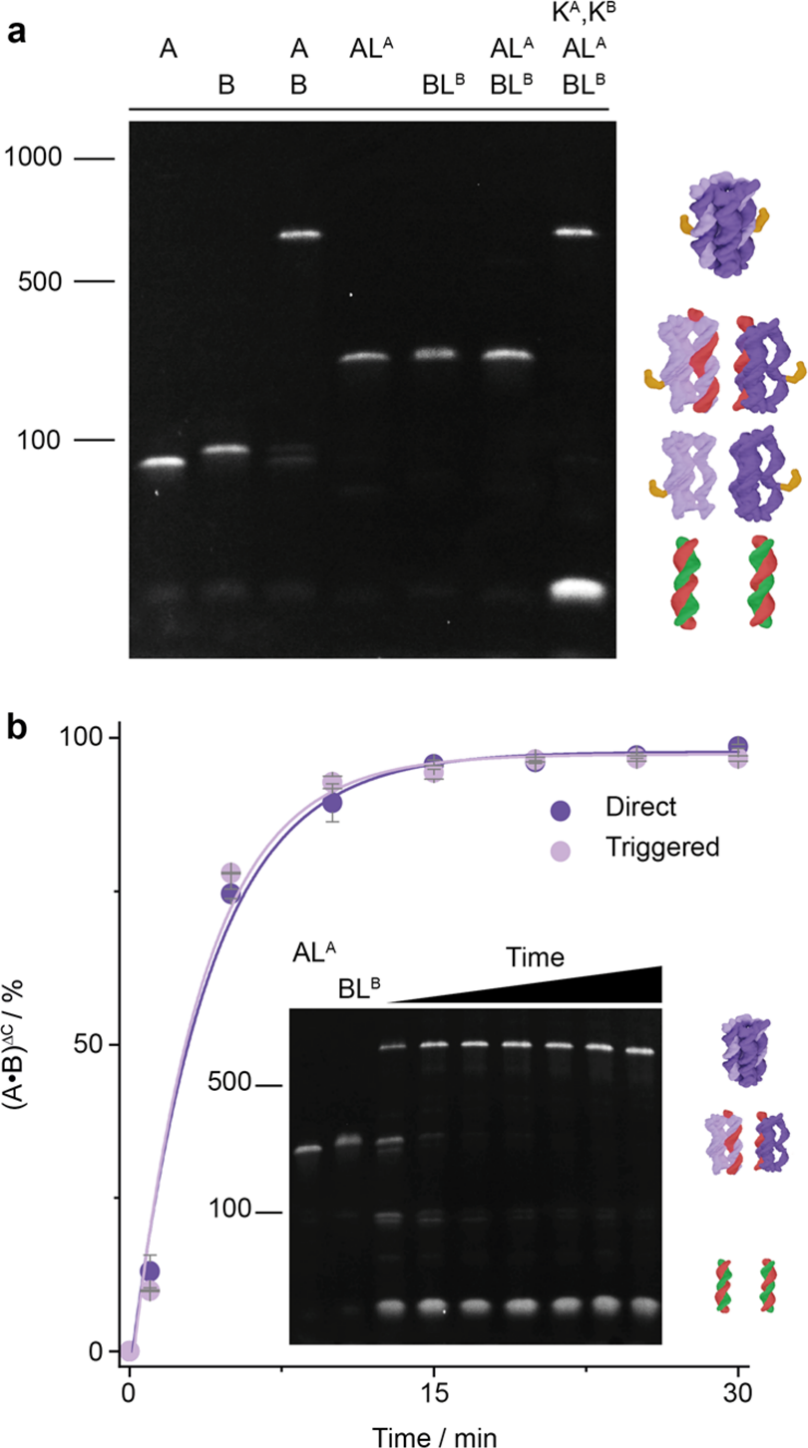
Nanopore A•B forms by direct and triggered assembly. (a) PAGE analysis of A•B formation by direct assembly from components A and B and triggered assembly of AL^A^ and BL^B^ in either the absence or presence of key strand K^A^ and K^B^. (b) PAGE-based kinetic analysis of (A•B)^ΔC^ formation by direct (dark purple) and triggered (light purple) mechanisms. A representative PAGE gel for triggered assembly is shown as an inset.

Direct pore assembly was first assessed in solution. Using polyacrylamide gel electrophoresis (PAGE) as a read-out, isolated components A and B appeared as fast migrating single bands (Figure 2a, lanes A and B), implying a homogeneous population. By comparison, the mixing of A and B (Figure 2a, lane A / B) resulted in a significant band shift, indicating the formation of the larger, assembled 4-duplex pore A•B (Figure 2a, lane A / B). The lack of other significant bands suggests that pore assembly was quantitative.

Pore formation also proceeded via triggered assembly in solution. The two components with locks, AL^A^ and BL^B^ (Figure 2a, lanes AL^A^ and BL^B^), migrated higher than components A and B without locks, as expected for constructs of greater masses. In further agreement, when mixed, the assembly locked components AL^A^ and BL^B^ migrated without additional bands that would indicate interaction (Figure 2a, lane AL^A^ / BL^B^). The addition of stoichiometric amounts of the keys K^A^ and K^B^, however, resulted in the complete removal of AL^A^ and BL^B^ bands and the concomitant formation of a higher migrating A•B band (Figure 2a, rightmost lane) that matched the band from direct pore formation. The assembly byproducts, duplexes L^A^K^A^ and L^B^K^B^, migrate to the bottom of the gel (Figure 2a, rightmost lane). Pore assembly was further demonstrated with cholesterol-free components A^ΔC^ and B^ΔC^ and the corresponding locked components A^ΔC^L^A^ and B^ΔC^L^B^ (Figures S3 and S4). The data show that pore formation in solution is not influenced by the absence or presence of the cholesterol tags. The equivalence of the pore formed by direct assembly compared to an annealed control was also confirmed with UV-thermal melting analysis. The melting profiles of a mixture of A^ΔC^ + B^ΔC^ and preannealed (A•B)^ΔC^ were very close (Figure S5a) resulting in identical melting temperatures (*T*_m_) of 63.1 ± 0.1 and 63.1 ± 0.2 °C, respectively (*n* = 3, Figure S5b).

Following the successful confirmation of triggered pore assembly, we investigated the effect of varying the key concentration on assembly. The addition of the key, K^A^, to the corresponding assembly locked component, A^ΔC^L^A^, led to the expected component unlocking with no other interactions even at a 10-fold excess of the key (Figure S4a). When the noncomplementary key, K^B^, was added to A^ΔC^L^A^, the ternary complex A^ΔC^L^A^K^B^ was formed (Figure S4b). In this complex, K^B^ is bound to component A^ΔC^ given their partial sequence complementaries. This is an implicit consequence of designing a trigger-assembled duplex bundle. The same is true for the binding of K^A^ and B^ΔC^L^B^ (data not shown). In a further experiment, both keys were added at increasing concentrations to both assembly locked components (Figure S4c). As previously demonstrated, a 1:1 ratio of key:assembly locked component led to quantitative pore formation. However, higher ratios of both keys increasingly inhibited the pore assembly as an implied consequence of the design of the DNA trigger-assembled pore. The side products were the ternary complexes A^ΔC^L^A^K^B^ and B^ΔC^L^B^K^A^ where the bound mismatched key blocks assembly to the pore. The experiment also led to the formation of a new complex which ran slightly above the band for the folded pore (A•B)^ΔC^ (Figure S4c). The new complex is an incompletely folded, open barrel-like structure in which bound keys prevent assembly into the 4-duplex pore (Figure S4c).

### Affinity and Kinetics of Pore Assembly in Solution

After confirming pore assembly, we determined the equilibrium dissociation constant, *K*_d_, and the kinetic rate constant, *k*_on_. We first used an electrophoretic mobility shift assay (EMSA) to derive *K*_d_. Visually tracking pore formation over a range of ratios of A^ΔC^:B^ΔC^ resulted in the expected binding profile (Figure S6a,b) and 1:1 binding stoichiometry. Plotting the gel band intensities (Figure S6a,b) yielded a Langmuir-derived *K*_d_ of 154 ± 23 nM (*n* = 3).

As the EMSA-derived *K*_d_ may be influenced by the limited sensitivity of ethidium bromide staining in gel electrophoresis, we used the more sensitive detection method of Förster resonance energy transfer (FRET). For this analysis, components A^ΔC^ and B^ΔC^ were labeled with FRET donor dye Cy3 and acceptor dye Cy5, respectively. Component mixing led to the expected FRET signal when the dyes are proximal upon pore formation. In particular, the Cy3 emission at 563 nm was reduced, and the Cy5 emission at 670 nm was increased (Figure S7a). A quantitative analysis of the fluorescence signals after titrating ^Cy5^B^ΔC^ over constant ^Cy3^A^ΔC^ using the same ratios as used for EMSA but at a 2.5-fold lower concentration yielded a *K*_d_ of 53.1 ± 5.4 nM (*n* = 3, Figure S7a,b, Table 1).

**Figure 3:**
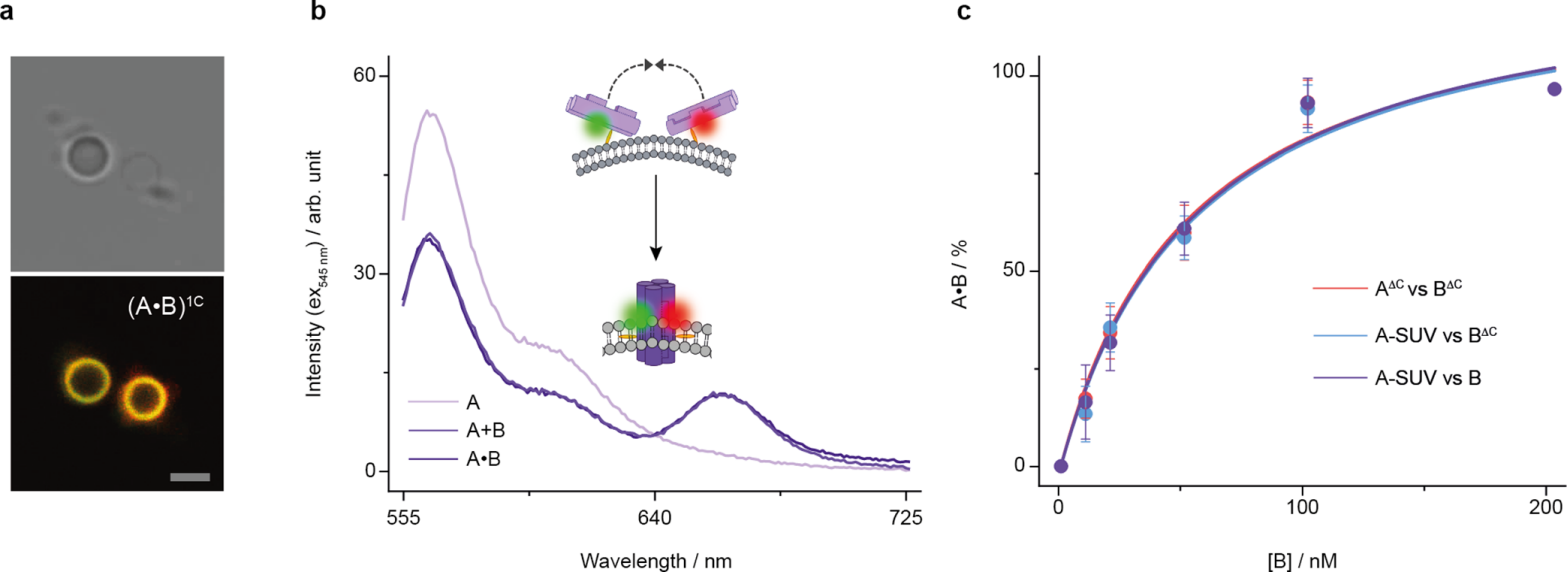
Assembly of DNA nanopore A•B from fluorophore-labeled components at the membrane interface. (a) Confocal microscopy images of the tethering of (A•B)^1C^ to a GUV composed of POPC lipids following incubation with ^Cy3^A and ^Cy5^B^ΔC^. Bright-field image (top) and overlay (bottom) of Cy5 (red) and Cy3 (green) channels. Scale bar, 5 μm. (b) FRET analysis and emission spectra for pore assembly of ^Cy3^A and ^Cy5^B on SUVs composed of DPhPC lipids, using excitation at 545 nm. The ^Cy3^A emission peak at 563 nm (pink) drops when ^Cy5^B is added at a 1:1 ratio (purple) to the same level as for the preannealed A•B pore (dark purple). (c) Concentration-dependent Cy3-emission change indicating pore assembly in solution (A^ΔC^ vs B^ΔC^) and at the membrane interface (A-SUV vs B^ΔC^, and A-SUV vs B) (*n* = 3).

The FRET-derived *K*_d_ was corroborated using dual-color fluorescence cross-correlation spectroscopy (FCCS).^55−57^ Using a 10-fold lower concentration range than in FRET, FCCS measurements led to a *K*_d_ of 62.2 ± 12.5 nM (*n* = 3, Figure S8b) which is within error of the *K*_d_ obtained from FRET (Table 1). Dropping the concentrations of the labeled components further 5-fold did not lead to saturation in binding (Figure S8a). In a further analysis, FCCS did not indicate any significant difference between the binding strength of either component; when the concentration of B^ΔC^ was held constant while A^ΔC^ was varied, the *K*_d_ was 71.7 ± 7.7 nM (*n* = 2) (Figure S8a) which is within error of the other *K*_d_. The *K*_d_ is more than an order of magnitude higher than the affinity obtained for other DNA duplexes of comparable length.^58^ This likely reflects the molecular difference between sterically restrained duplex formation from three component strands within the DNA nanopore and binding of two conformationally unlocked single stranded DNA strands.

**Table 1:** Summary of Equilibrium and Kinetic Data for the Assembly of Nanopore A•B from Components A and B in Solution (A^ΔC^ + B^ΔC^), at the Membrane Interface (A-SUV + B^ΔC^), and on the Membrane Surface (A-SUV + B) Using Förster Resonance Energy Transfer (FRET)

parameter	condition	av ± SD^a^
*K*_d_ (M)	A^ΔC^ vs B^ΔC^	5.3 ± 0.5 × 10^–8^
	A-SUV vs B^ΔC^	5.6 ± 0.2 × 10^–8^
	A-SUV vs B	5.6 ± 0.6 × 10^–8^
*k*_on_^b^ (M^–1^ s^–1^)	A^ΔC^ + B^ΔC^	11.9 ± 2.8 × 10^3^
	A-SUV + B^ΔC^	5.9 ± 0.6 × 10^3^
	A-SUV + B	1.9 ± 0.3 × 10^3^
*k*_off_ (s^–1^)	A^ΔC^ + B^ΔC^	6.3 ± 1.9 × 10^–4^
	A-SUV + B^ΔC^	3.1 ± 0.4 × 10^–4^
	A-SUV + B	1.1 ± 0.2 × 10^–4^

a Averages and standard deviations were obtained from at least three independent repeats.

b *k*_on_ derived from kinetic trace with initial fluorescence drop removed.

After determining *K*_d_, we measured the kinetic rate constant, *k*_on_, of pore assembly. Using EMSA, we examined whether the kinetics of triggered assembly are different to those of direct assembly. The *k*_on_ values obtained for direct assembly (4.5 ± 0.4 × 10^3^ M^–1^ s^–1^, *n* = 3, Figure 2b, Figure S9a,b) and triggered assembly (4.7 ± 0.5 × 10^3^ M^–1^ s^–1^, *n* = 3; Figure 2b, inset; Figure S9c,d) were within error. This indicates that the triggering mechanism is not rate-limiting.

We confirmed the EMSA-derived kinetic data with a FRET-based continuous kinetic assay. The time-dependence of the FRET signal after component mixing yielded a *k*_on_ of 11.9 ± 2.8 × 10^3^ M^–1^ s^–1^ (*n* = 3, Table 1, Figure S10), which is close to the EMSA-derived value. The rate constants at around 10^3^ M^–1^ s^–1^ are 2–3 orders of magnitude slower than typical DNA duplex hybridization.^59,60^ Similar to the weak *K*_d_, the molecular reasons for the slower kinetics are likely a result of slow nucleation due to the presence of additional sequences of the nontarget arms and loops, and slow zippering due to a significant secondary structure resulting from arm and loop interactions.^61−64^

### Pore Assembly at the Membrane Interface

After characterizing pore formation in solution, we investigated pore assembly at the membrane interface. We first incubated cholesterol-tagged ^Cy3^A with giant unilamellar vesicles (GUVs) and then added the non-cholesterol-modified ^Cy5^B^ΔC^ and detected the lipid-anchor-mediated membrane tethering using confocal microscopy. Overlapping Cy3 and Cy5 fluorescent halos around the GUVs suggest that the two components assembled into pore (A•B)^1C^ at the membrane interface (Figure 3a and Figure S11).

To obtain quantitative information on pore assembly at the membrane, we used a FRET assay using small unilamellar vesicles (SUVs). As a baseline, we first added ^Cy3^A to SUVs and acquired a Cy3 emission spectrum (Figure 3b, pink; Figure S7). Adding the second component, ^Cy5^B, to membrane-anchored ^Cy3^A in a 1:1 ratio resulted in a FRET-induced decrease in Cy3 and an increase in Cy5 emissions, respectively, implying pore assembly (Figure 3b, purple). The emission spectrum overlapped with a control trace for an A•B pore that was preannealed before addition to SUVs (Figure 3b, dark purple; Figure S7). The spectral equivalence indicates quantitative assembly on the membrane surface. Part of the change in fluorescence is likely caused by the proximity of the fluorophores when ^Cy3^A and ^Cy5^B are tethered to the membrane but not assembled. This contribution can be seen by a weak FRET change upon adding assembly-inactive ^Cy3^AL^A^ and ^Cy5^BL^B^ in a 1:1 ratio to the SUVs, which resulted in a FRET change equivalent to 32.0 ± 3.9% assembly (*n* = 3, Figure S12a). By contrast, mixing of ^Cy3^A and ^Cy5^B resulted in a signal change of 96.3 ± 6.6% relative to the preannealed control confirming pore assembly (Figure 3c, Figure S12b).

To additionally probe for the insertion of A•B pores into SUV membranes, we analyzed the melting profiles, as bilayer insertion is known to confer increased stability to DNA pores and a higher *T*_m_.^32,65^ The *T*_m_ values for A•B assembled on the membrane and preannealed prior to SUV incubation were 3 °C higher than for the non-SUV sample (Figure S4) implying membrane insertion.

### Affinity and Kinetics of Pore Assembly at Membranes

We obtained the equilibrium dissociation constant, *K*_d_, for pore formation at the bilayer interface, by adding component ^Cy5^B to ^Cy3^A-anchored SUVs and measuring the change in FRET. Plotting the normalized FRET signal as a function of ^Cy5^B concentration (Figure S7e,f) led to a *K*_d_ of 55.8 ± 6.2 nM (*n* = 3, Table 1). This is within error of the *K*_d_ values for pore formation in solution as well as for the assembly of cholesterol-free ^Cy5^B^ΔC^ with SUV-bound ^Cy3^A (Table 1, Figure S7a–d). In agreement, yields for pore assembly obtained from FRET efficiency (*E*) calculations revealed that pore assembly occurs in high yield across all three conditions (Table S3). A similar equivalence of affinity values in pore formation was obtained using EMSA analysis (*K*_d_ = 1.64 ± 0.14 × 10^–7^ M, *n* = 3; Figure S6c,d).

A kinetic FRET analysis of ^Cy3^A and ^Cy5^B assembly on the membrane (Figure S10) revealed an association rate constant, *k*_on_, of 1.9 ± 0.3 × 10^3^ M^–1^ s^–1^ (*n* = 3), which is 1 order of magnitude slower than in solution (Table 1). Likely, pore formation on the membrane surface is sterically hindered by membrane anchoring. We note that the *k*_on_ value is corrected for an initial sharp drop in Cy3 emission when ^Cy5^B is mixed with vesicles carrying membrane-anchored ^Cy3^A (Figure S10a). It occurs most likely because the rapid binding of ^Cy5^B to the membrane leads to the close proximity to ^Cy3^A and a weak FRET signal. As support of this interpretation, the extent of the rapid initial drop, obtained via normalized intensity (*F*/*F*_0_), was 0.91 ± 0.03 (Figure S10a), which is close to the value of 0.92 ± 0.04 (Figure S12) for the previously discussed assembly blocked AL^A^ + BL^B^.

The experimentally determined *K*_d_ and *k*_on_ were used to calculate the dissociation rate constant, *k*_off_, using eq 1 assuming a second-order system:
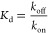
1

The *k*_off_ for pore assembly at membranes is 1.94 ± 0.53 × 10^–4^ s^–1^, which is 2–3 orders of magnitude slower than typical values for simple DNA hybridization that range between 10^–1^ and 10^–3^ s^–1^ (refs (61−63)). The lower *k*_off_ is plausible given the required multiple duplex dissociations to separate the pore into its two components. Other contributions come from the movement of the separated components against the lateral membrane pressure and repositioning of the separated pore components from a membrane-spanning to tethering orientation. The quantitative kinetic analysis was complemented by visually tracking the pore assembly on supported lipid bilayers using single-molecule FRET (smFRET) and single-particle tracking (Figure S13). The analysis yielded a FRET efficiency of 0.39 ± 0.3 very close to the values found for assembly on the vesicle membranes (Table S3).

### Probing the Influence of Steric Factors on Hybridization

The kinetic analysis of A•B pore formation at membranes revealed that *k*_on_ and *k*_off_ are strongly different to solution. The likely reason is that duplex formation and dissociation require the DNA components to change their position from a membrane-adhering to membrane-spanning state, and back, respectively. We sought to corroborate this theory with a model system where DNA hybridization is taking place outside the membrane and hence is expected to be less influenced by steric factors. The model was based on DNA duplex hybridization of a 20 nt DNA strand, S, to a complementary strand, R (Figures S14 and S15, Tables S4 and S5). To probe for the influence of steric bulk, S is optionally carrying a six-duplex DNA barrel of 15.5 × 5.5 nm while R is optionally cholesterol-anchored to SUV membranes (Tables S4 and S5). Hybridization was assessed for all conditions using EMSA and FRET (Figures S16–S19). In line with expectations, an analysis of DNA hybridization by EMSA (Figures S16 and S18) revealed that *K*_d_ is largely unaffected by the absence or presence of the nanobarrel (Table S6 and Figures S16 and S18). Membrane anchoring led, however, to weaker affinity, but not in the presence of the nanobarrel where the affinity was unaffected.

Similarly, *k*_on_ values of the duplex model were not largely influenced by bulk or membrane anchoring (Table S6 and Figures S19 and S20). The values were also in line with literature studies on DNA duplex formation.^59,60,62,66,67^ This suggests that the slower kinetics for the A•B pore assembly are to a large extent a consequence of the previously discussed slow nucleation and zippering steps. The duplex insertion into the bilayer also likely has an effect on the kinetics.As a further insight, the FRET-derived extent of assembly for the duplex model dropped by ∼40% at the membrane interface compared to solution (Table S7), consistent with previous reports,^59,64,68,69^ while the yields of highly favorable A•B pore assembly was not significantly affected by the membrane (Table S3)..

### Investigating the Orientation of A•B at the Membrane Interface

We first probed the orientation of A•B relative to the bilayer membrane using a nuclease digestion assay^70^ (Figure S21). In the assay, membrane-inserted DNA pores are partly protected from digestion by the nuclease compared to more sterically accessible DNA pores in solution. Indeed, solvated pores with zero, one, and two cholesterols, (A•B)^ΔC^, (A•B)^1C^, and A•B, respectively, were rapidly and completely digested (Figure S21). By comparison, incubating pores with large unilamellar vesicles (LUVs) prior to nuclease incubation led to the anticipated cholesterol-dependent reduction in both the rate and extent of digestion (Figure S21). While noncholesterol and nonbinding (A•B)^ΔC^ was unaffected, (A•B)^1C^ gained protection (Figure S21) as the single cholesterol is expected to tether the pore parallel to the LUV membrane (Figure 1, Figure 3b) and render it less accessible to the nuclease. Double cholesterol-tagged A•B experienced the biggest nuclease protection (Figure S21) in line for a pore that is expected to span the lipid bilayer (Figures 1 and 3b). These measurements do not elucidate which percentage of (A•B) pores span the membrane.

**Figure 4:**
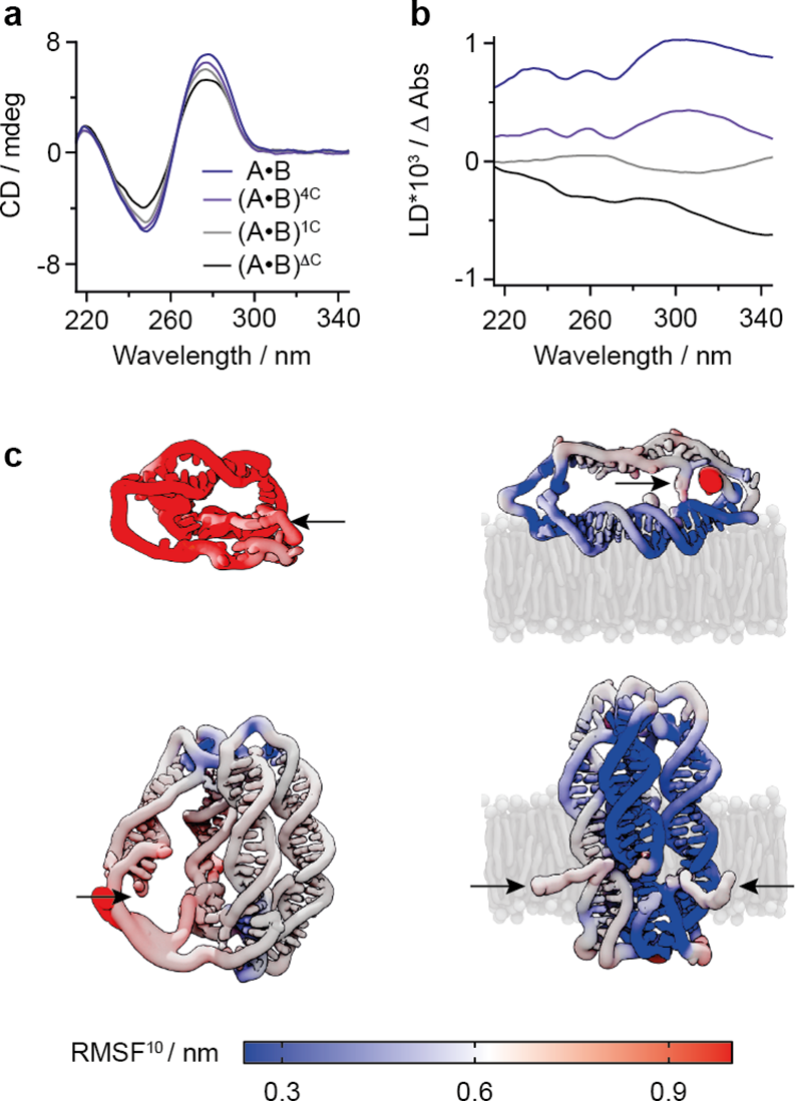
Stability and dynamics of membrane-tethered and membrane-spanning DNA nanostructures. (a) Representative CD spectra of A•B (dark purple) and controls (A•B)^4C^ (purple), (A•B)^1C^ (gray), and (A•B)^ΔC^ (black). (b) Representative LD spectra of A•B (dark purple), (A•B)^4C^ (purple), (A•B)^1C^ (gray), and (A•B)^ΔC^ (black) in the presence of SUVs composed of POPC. (c) Representative snapshots of the DNA nanostructures from four simulated trajectories. Component A in solution (top-left) and tethered to a membrane (top-right), and A•B in solution (bottom-left) and spanning a membrane (bottom-right). The simulated POPC bilayer is shown in gray, and solvent atoms are omitted for clarity. DNA nanostructures are colored by the per-residue RMSF^10^ to indicate regions of structural flexibility (red = high, white = med, blue = low). The location of the hydrophobic cholesterol anchors is indicated with black arrows.

To corroborate the cholesterol-dependent orientation of our DNA nanopore, we used dichroism spectroscopy. Using circular dichroism (CD) spectroscopy, we first ascertained the helical structure of the nanopore. The CD spectra of A•B variant constructs with either 4, 1, or no cholesterols in the absence of membranes exhibited the characteristic signature for the expected B-form DNA with a negative peak at 245 nm and a positive peak at 280 nm (Figure 4a).^71,72^ The lack of competing peaks in the CD spectra suggests that all constructs form typical B-type helical structures.

Linear dichroism (LD) was then used to probe the orientation of the A•B pore relative to SUV membranes. A positive peak at 260 nm in the LD spectra indicated that A•B with two cholesterol anchors (Figure 4b, dark purple) was oriented perpendicular to the bilayer,^73,74^ indicative of a membrane-spanning orientation. In further agreement, pore variant (A•B)^4C^ with four cholesterols yielded a similar spectrum (Figure 4b, purple). The spectrum of control pore (A•B)^ΔC^ without cholesterol featured a flattened spectral line (Figure 4b, black), as expected for a lack of membrane interaction. The broad negative LD peak for (A•B)^1C^ with one cholesterol (Figure 4b, gray) suggests a dynamic orientation parallel to the bilayer.^74^ The noisy nature of the spectra is assumed to be in part due to the effects of light scattering from the SUVs with diameters of ∼180 nm.^74−76^

### Molecular Dynamics Simulations Provide Insight into the Structural Dynamics of Pore Components and the Pore

The membrane-dependent interactions of component A and assembled A•B were further investigated using atomistic molecular dynamics. Insight from experimental data was used to inform the initial configurations of the simulated trajectories. Structural dynamics were investigated using the per-residue root-mean-square fluctuation (RMSF^10^) (Figures S22–S24). The baseline analysis of component A in solution yielded a highly labile structure, with an average regional fluctuation of 0.72 ± 0.38 nm (Figure 4c, top-left; Figure S24). The addition of a lipid bilayer membrane, however, stabilized component A to an average region fluctuation of 0.49 ± 0.17 nm (Figure 4c, top-right; Figure S24). The stabilization is likely caused by cholesterol tag-mediated proximity of the DNA nanostructure to the membrane resulting in electrostatic interactions between the DNA duplex and lipid headgroups (Figure S25).

In comparison to component A, pore A•B in solution was significantly more stable yet remained dynamic with an average regional RMSF^10^ of 0.42 ± 0.14 nm (Figure 4c, Figures S23 and S24). The simulations also indicate that the nicks in each duplex are a hinge-point (Figure 4c, Figure S1), and that the TEG-cholesterol groups are highly mobile and coil inside the attached or neighboring duplex (Video S1), consistent with previous reports.^77^ By contrast, membrane interaction stabilized the A•B pore resulting in a lowered regional averaged RMSF^10^ of 0.35 ± 0.15 nm and a compact pore geometry (Figure 4c, Figure S24). As a likely reason for stabilization, the lipid bilayer reduces the structural flexibility of the pore’s central lumen and strand breaks.

Increased stabilization was also found for one of the noncholesterol duplexes (RMSF^10^ of 0.2–0.3 nm) positioned between two cholesterol-modified duplexes (RMSF^10^ of 0.3–04 nm) (Figure 4c, Figure S23). This stabilization is likely due to linker-cholesterol groups which are positioned close to the unmodified duplexes and thereby reduce the dynamics of the surrounding phospholipid molecules.

We corroborated the membrane-induced changes from a globular structure to a compact pore by comparison with the average intrafluorophore distance of the Cy3–Cy5 on A•B (Table S8), which is a useful proxy for pore diameter. The Cy3–Cy5 distances were derived from the FRET efficiency as described.^78^ The suggested membrane-induced compression of the inserted pore was supported by a slight drop in the intrafluorophore distances from 7.10 ± 0.50 nm for the non-membrane-spanning pore (A•B)^ΔC^ to 6.63 ± 0.15 nm for an A•B pore within SUVs. In agreement, control pore (A•B)^1C^ in a membrane-tethered but not compressing state remained at the solution-phase distance of 7.05 ± 0.14 nm.

### Membrane–Pore Interactions Alter the Lipid Bilayer Structure and Dynamics

**Figure 5:**
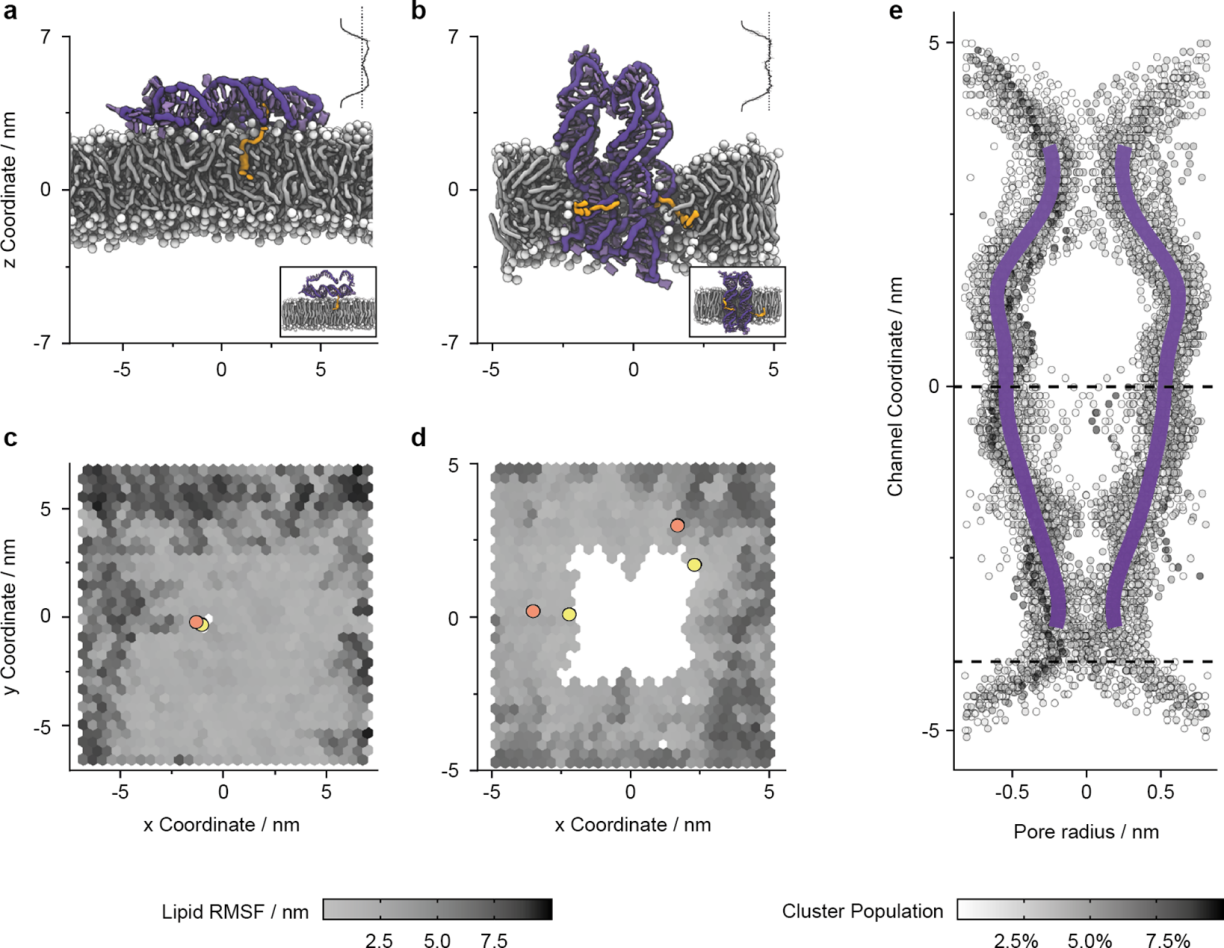
Membrane anchoring of component A and nanopore A•B affects the lipid bilayer as analyzed with molecular dynamics simulations. Representative snapshots of the equilibrated regions of trajectories of (a) membrane-tethered component A and (b) membrane-spanning A•B. The insets show the initial configurations. The DNA is colored purple, and the TEG-cholesterol linkers are orange-yellow. The POPC bilayer is colored white (headgroups) and gray (lipid tails). Solvent is omitted for clarity. The average bilayer densities from A and A•B trajectories are plotted in the upper-right of each snapshot, with a dashed line indicating the density of the membrane at this midsection in the absence of a nanopore (0.425 g/(mol A^3^)). (c, d) Top-down views corresponding to panels a and b, respectively, with DNA omitted for clarity. Each hexagon is colored by the per-molecule-lipid RMSF (black = low, white = high). The locations of the cholesterol lipid anchors are indicated at the start (orange) and end (yellow) of the trajectory. (e) Channel lumen mapping using HOLE^80^ analysis on a series of clustered simulation snapshots of the A•B channel in a POPC bilayer in 1 M KCl. The proportion of the trajectory represented by each cluster is indicated by the transparency of the points, and a trend line has been plotted to estimate the average channel shape. The two black dashed lines represent the approximate positions of the bilayer headgroups relative to the nanopore channel coordinate in the most populated cluster.

MD simulations were also used to assess if and to what extent membrane-interacting component A and pore A•B altered the lipid bilayer structure and dynamics. Following the simulations, tethering of component A to the membrane resulted in minimal structural changes to the bilayer (Figure 5a) but component A was flattened on the membrane surface (Figure 5c, Figure S25).

In contrast, the membrane-spanning orientation of A•B resulted in significant lipid remodeling by forming a toroidal lipid arrangement surrounding the pore perimeter (Figure 5b, inset shows initial state; Video S2) consistent with previous modeling.^46,79^ The toroidal arrangement positions the lipid headgroups next to the DNA nanopore and thereby shields the hydrophobic membrane core from the hydrophilic charged DNA helices (Figures S26 and S27). This caused a reduction in the average bilayer density nanopore interface and (Table S9).^48^ The formation of the toroidal lipid arrangement was also accompanied by the alignment of the cholesterol anchors parallel to the fatty acid tail of the phospholipids (Figure 5b, Video S2) and an upward movement of A•B relative to the bilayer plane (Figure S28). Cholesterol moieties stabilized the surrounding bilayer as indicated by the reduced RMSF near the lipid anchors (Figure 5d).

### Mapping of the Channel Lumen Using MD Simulations

We used MD simulations to elucidate the shape of the channel lumen. Cluster analysis was performed on the transmembrane A•B pore trajectory to generate a series of coordinates, which were then analyzed using the HOLE^80^ software (Figure 5e, Figure S29). The simulated map indicates that the pore has a dynamically changing lumen^44^ at the midsection that ranges in diameter from 0.60 ± 0.19 to 1.02 ± 0.18 nm with an overall average of 0.83 ± 0.14 nm. The pore is narrower at the top and bottom where the component duplexes are cross-linked. Nevertheless, these two regions also showed variation in diameter (Video S2). A third narrowing of the channel is observed at the position of the cholesterol tags likely due to the bilayer-induced dynamic compression.

### Triggered Assembly of A•B on the Membrane Surface Results in a Functional Nanopore

**Figure 6:**
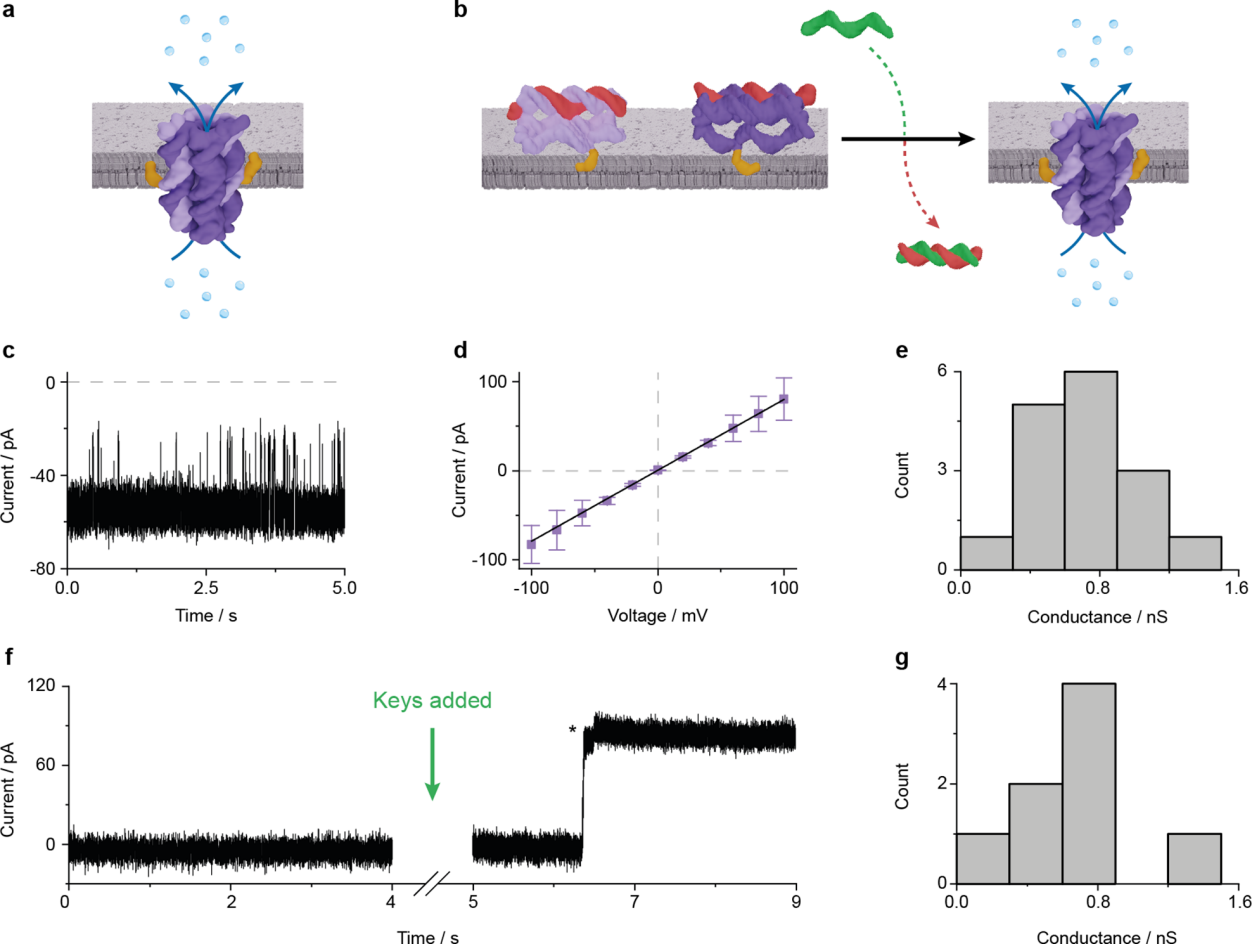
Characterization of the conductance properties of the nanopore A•B. (a) Schematic illustration of a preassembled A•B inserted into a planar lipid bilayer. (b) Schematic illustration of triggered assembly at the membrane interface, induced by the addition of key strands. (c) Representative ionic current traces from a single A•B nanopore inserted in a planar lipid bilayer composed of DPhPC lipids in 1 M KCl, 10 mM HEPES, pH 7.4, at a membrane potential of −50 mV. (d) Current–voltage relationship for voltages ranging from −100 to +100 mV at 20 mV steps, showing ohmic properties of the A•B channel and displaying averages ± SEM from 16 independent pore insertions. (e) Histogram of channel conductance obtained from 16 independent single-channel recordings at membrane potentials ranging from +20 to +50 mV. (f) Representative ionic current trace showing how the addition of the key strands to membranes containing AL^A^ and BL^B^ results in the formation of nanopore A•B at +90 mV. The asterisk indicates a small current step likely caused by molecular changes in the pore structure upon complete assembly. (g) Histogram of channel conductance obtained from 8 independent single-channel recordings of trigger-assembled nanopores at membrane potentials of +20 mV.

Following characterization of pore formation and its interaction with membranes, we endeavoured to characterize pore activity. In particular, we were interested in determining whether A•B formed at the membrane functioned as a bilayer-spanning nanopore (Figure 6a,b). This question was addressed with single-channel current recordings (SCCRs). Preannealed A•B was examined first to establish the reference for the conductance of single nanopores. The addition of A•B to planar lipid bilayers resulted in a change in current to −54 pA at −50 mV (Figure 6c). The current was recorded as a function of voltage for 16 independent insertions to confirm the presence of DNA pores. The ohmic current–voltage dependence between ±100 mV (Figure 6d) matched the signal expected for the vertically symmetrical A•B as also found for comparable DNA nanopores.^32,65,81^ The analysis also yielded a mean conductance of 0.70 ± 0.27 nS (Figure 6e) which is consistent with a theoretical conductance of 0.67 nS based on a nominal lumen diameter of 0.8 nm obtained from the lumen analysis by MD simulations (Figure 5d).

After characterizing preannealed A•B, we investigated whether triggered A•B assembly at the membrane surface resulted in pore characteristics comparable to those of the directly assembled pore. For this investigation, the assembly locked components AL^A^ and BL^B^ were incubated with planar lipid bilayer membranes. This did not lead to a current change indicating a lack of membrane puncturing (Figure 6f). However, the addition of keys K^A^ and K^B^ led to pore assembly on the membrane surface as confirmed by the characteristic increase in current to 71 pA at +90 mV (Figure 6f) consistent with membrane inserted A•B. A small additional current step (Figure 6f, asterisk) of 4.0 pA suggests small-scale rearrangements of the duplexes upon pore insertion into the lipid bilayers. A further analysis from 8 independent insertions of trigger-assembled pores yielded a mean pore conductance of 0.69 ± 0.12 nS (Figure 6g) which is within error of the preassembled pore. The electrical recordings confirm that the triggered assembly of A•B upon addition of keys results in a functionally identical nanopore to the directly assembled pore.

### A•B Transports Molecular Cargo across Lipid Bilayers

**Figure 7:**
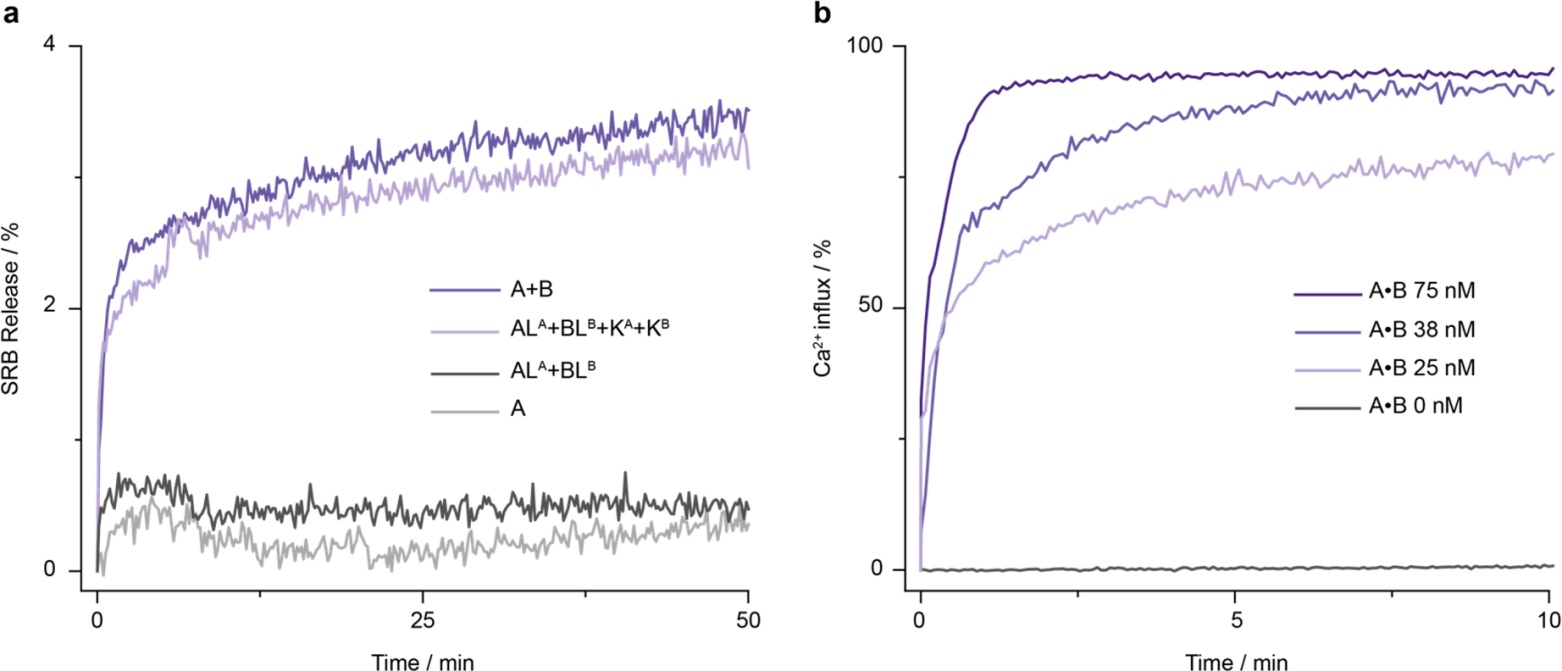
Transport-active A•B channels are formed by assembly from inactive components. (a) Kinetic fluorescence traces for sulforhodamine B (SRB) efflux mediated by direct assembly of A and B and triggered assembly of A•B from inactive subunits AL^A^ and BL^B^. The fluorescence signal originates from SRB that is contact-quenched at high concentrations within DPhPC SUVs but regains its emission when released at low concentrations into the ambient. A signal of 100% is obtained by rupturing the SUVs by the addition of detergent. Each trace is the average of three independent repeats obtained at 400 nM. (b) Kinetic fluorescence traces on Ca^2+^ influx across membrane-inserted (A•B)^4C^ into POPC SUVs containing the Ca^2+^-sensitive dye Fura-2. The number of repeats for traces at 75, 38, 25, and 0 nM are 3, 2, 1, and 3, respectively. Maximum influx was recorded following the addition of a detergent to rupture the vesicles.

According to the MD simulations and SCCR experiments, the central pore lumen width of around 0.8 nm should support the flux of small-molecule cargo. To investigate this, we probed molecular transport across the A•B pore formed via direct assembly from components A and B, or via triggered assembly from locked components AL^A^ and BL^B^ and the addition of unlocking keys K^A^ and K^B^. In the transport assay, the fluorophore sulforhodamine B (SRB) is encapsulated inside SUVs where it is contact quenched but increases in brightness when it effluxes across membrane pores into the ambient.^32,51,81^ As expected, there was no dye flux when the SRB-filled SUVs were incubated with individual components or the assembly locked components in the absence of keys (Figure 7a, Figure S30).

Adding directly assembled and trigger-assembled A•B at 400 nM to vesicles resulted in very similar dye effluxes (3.57 ± 0.14% and 3.33 ± 0.26%, respectively) (Figure 7a) and lower fluxes when 200 nM pores were used (Figure S30). The flux was 5.25 ± 1.05% when 400 nM preannealed pore A•B was used. The data indicate that the *in situ* assembled A•B results in a functional nanopore. The transport at around 3–5% is low, and this is consistent with the expected slow diffusion of SRB (diameter ∼0.7 nm^32^) across a pore with a narrow lumen 0.8 nm in diameter.

### A•B Forms Synthetic Ca^2+^ Permeable Channels

The dye flux assay indicated that the narrow lumen of A•B makes it more suitable to the transport of cargo smaller than a fluorescence dye. Ca^2+^ with its small size (0.23 nm, ref (82)) and positive chargewas expected to transport more efficiently across the pore than SRB and thereby complement the SCCR data on ion transport. In the transport assay,^83,84^ the Ca^2+^-sensitive ratiometric dye, Fura-2, was encapsulated at 100 μM within SUVs, and 250 μM CaCl_2_ was added to the ambient fluid. In the absence of pores, the SUV membranes were impermeable to Ca^2+^ as confirmed by fluorescence analysis (Figure S31a). In addition, dynamic light scattering established that CaCl_2_ did not disrupt vesicle integrity or significantly alter vesicle diameter (Figure S31b). The addition of 25 nM preassembled pore (A•B)^4C^ resulted in considerable Ca^2+^ influx, reaching 79.2% signal after 10 min (Figure 7b). An even higher flux of 90.3 ± 3.1% (*n* = 3) was achieved with 75 nM (A•B)^4C^ after only 70 s. In the absence of pore, no significant Ca^2+^ influx was observed (0.94 ± 0.13%, *n* = 3).

## Conclusion

This study has pioneered the development of a synthetic DNA nanopore that forms by triggered assembly of inactive prepore components. Previous DNA pores were preformed in solution and integrated as complete pores into the membrane. The formation of the present pores proceeds either by direct assembly of the two pore subunits or via activating two assembly locked components with DNA keys that reactivate pore assembly. Both routes produce the same assembly yield and pore function. Controlled pore formation from DNA subunits and DNA triggers does not occur in nature. However, the concept is related to biological pores which assemble from membrane-tethered subunits. The oligomeric pores usually form via an intermediate non-membrane-spanning prepore state which matures to the membrane puncturing pore via spontaneous conformational changes, such as the α-hemolysin pore^85^ or by protease-triggered changes, often found in the membrane-attack complexes.^86^

The biomimetic DNA pore has provided insight into processes underpinning controlled pore formation. Analyzing the affinity and kinetics of nanopore assembly determined the influence of membranes on molecular interaction and assembly. DNA hybridization was slowed down by an order of magnitude because assembly of the pore subunits requires a change from a membrane-tethered to a membrane-spanning orientation. Conversely, dissociation of the pore into the subunits was slowed down as this requires a transition from the spanning to the tethered orientation which also reduces the structural flexibility of the DNA structures due to the stabilizing effect of the pore-surrounding lipid bilayer. As previous studies did not involve a similar change in DNA association or dissociation,^87^ we expect our findings to inspire the creation of dynamic functional nanostructures^88^ at the membrane interface and to contribute to a further understanding of molecular processes at membranes. By aiming to create synergies between chemistry and the life sciences,^89^ our study will help develop triggered DNA nanodevices for biomedicine, synthetic biology, and chemical biology.^90^

## Supporting Information Available

The Supporting Information is available free of charge on the ACS Publications Web site. SI (PDF) (Videos S1 and S2) The Supporting Information is available free of charge at https://pubs.acs.org/doi/10.1021/jacs.1c06598.

Experimental section and additional data and figures including 2D strand maps, schematics, PAGE characterization, gel electrophoretic mobility shift assays, UV melting profiles, and FRET analysis results (PDF)
Videos S1 and S2: Simulation of A•B interacting with a lipid bilayer (MOV, MOV)

## Supplementary Material

ja1c06598_si_001.pdf

ja1c06598_si_002.mov

ja1c06598_si_003.mov
