# Supplementary material for: Triggered Assembly of a DNA-Based Membrane Channel
Source: J Am Chem Soc. 2022 Mar 7;144(10):4333–44. doi: 10.1021/jacs.1c06598 (PMC8931747; doi:10.1021/jacs.1c06598)
Supplement: Supplementary file 1 — ja1c06598_si_001.pdf [file ja1c06598_si_001.pdf]

Supporting Information

# Triggered Assembly of a DNA-Based Membrane Channel

Conor Lanphere,[a] Jonah Ciccone,[a] Adam Dorey,[a] Nora Hagleitner-Ertuğrul,[b] Denis Knyazev,[b] Shozeb Haider,*[,c] Stefan Howorka*[,a]

[a] *Department of Chemistry, Institute of Structural Molecular Biology, University College London, London WC1H 0AJ, United Kingdom*

[b] *Institute of Applied Experimental Biophysics, Johannes Kepler Universität, 4040, Linz, Austria*

[c] *Department of Pharmaceutical and Biological Chemistry, University College London School of Pharmacy, London WC1N 1AX, United Kingdom*

# Table of Contents


**1. EXPERIMENTAL 5**

**1.1. Materials 5**

**1.2. DNA assembly 5**

**1.3. PAGE 5**

**1.4. Agarose gel electrophoresis 5**

**1.5. Preparation of small unilamellar vesicles (SUVs) 5**

**1.6. Melting temperature ($T_m$) analysis using UV-vis spectroscopy 6**

**1.7. Electrophoretic mobility shift assay 6**

**1.8. FRET assay on pore assembly 6**

**1.9. Dual-color FCCS analysis to measure the $K_d$ of pore assembly 7**

**1.10. Preparation of giant unilamellar vesicles (GUVs) 9**

**1.11. Confocal laser scanning microscopy 9**

**1.12. Preparation of planar lipid bilayers on a glass slide and smFRET and single particle tracking 9**

**1.13. Enzyme digestion assay 10**

**1.14. Circular dichroism 10**

**1.15. Linear dichroism 10**

**1.16. Simulation preparation 10**

**1.17. Simulation analysis 11**

**1.17.1. RMSF[10] 11**

**1.17.2. Clustering 12**

**1.17.3. Lumen analysis 12**

**1.17.4. Lipid analysis 12**

**1.18. Nanopore current recordings 12**

**1.19. Preparation of fluorophore-filled SUVs and dye release assay 12**

**1.20. Preparation of Fura-2-filled SUVs and $Ca^{2+}$ influx assay 13**

## 2. TABLES AND FIGURES 14

**Table S1. Names, modifications and sequences of DNA oligonucleotides used for folding A, B, A•B and variants. 14**

**Table S2. Names and strand compositions of structures used for the DNA nanopore with biomimetic triggered assembly 15**

**Figure S1. 2D strand maps of direct and triggered assembly of A•B. 16**

**Figure S2. Schematic illustrating the triggering mechanism and assembly in solution and on membranes. 17**

**Figure S3. PAGE characterization of A•B pore formation. 18**

**Figure S4. Gel electrophoretic mobility shift assay on the key specificity and the concentration dependence of triggered pore assembly 19**

**Figure S5. UV-melting profiles. 20**

**Figure S6. Gel electrophoretic mobility shift assay on the formation of the A•B pore. 21**

**Figure S7. Förster resonance energy transfer (FRET) analysis on pore assembly. 22**

**Figure S8. Dual-color fluorescence cross-correlation spectroscopy analysis of pore assembly. 23**

**Figure S9. PAGE kinetic electrophoretic mobility shift assays of direct and triggered pore assembly. 24**

**Figure S10. Pore assembly kinetics monitored via FRET. 25**

**Figure S11. Confocal microscopy images confirm cholesterol-mediated membrane tethering of (A•B)1C to GUVs and pore assembly on the membrane surface. 25**

**Figure S12. FRET control between assembly-locked components at the membrane surface. 26**

**Figure S13. smFRET and single particle tracking confirm A+B assembly at the membrane surface. 27**

**Table S3. Summarized FRET efficiency (E) data for pore assembly derived from the FRET pore assembly binding titrations and pore assembly kinetics. 27**

**Table S4. Names, modifications, and sequences of DNA oligonucleotides used for folding R, S and $S_{NP}$. 28**

**Table S5. Names and strand compositions of structures used for the model system. 29**

**Figure S14. Schematic illustration of the model system used to investigate the effect of sterics on DNA hybridization. 30**

**Figure S15. Structure and assembly confirmation of $S_{NP}$. 31**

**Figure S16. Gel electrophoretic mobility shift assay to assess the binding of the receptor (R) to the ssDNA ligand (S) or nanopore-bound ligand ($S_{NP}$).** **32**

**Figure S17. Fluorescence analysis of the interaction of R and SNP labeled with the FRET pair, Cy3 and Cy5, respectively.** **33**

**Figure S18. Gel electrophoretic mobility shift assay to assess the binding of the ssDNA ligand (S) or nanopore-bound ligand ($S_{NP}$) to the receptor (R), which is tethered to the surface of SUVs.** **34**

**Figure S19. FRET kinetic analysis of hybridization between R and S (or $S_{NP}$) in solution and on the membrane surface.** **35**

**Figure S20. Confocal microscopy images of hybridization between $S_{NP}$ and R on the surface of GUVs.** **36**

**Table S6: Summary of thermodynamic and kinetic data obtained for the four conditions of the model system.** **37**

**Table S7. Summary of FRET efficiencies derived from the hybridization kinetics between the four components of the model system.** **37**

**Figure S21. Digestion assay to confirm pore insertion.** **38**

**Figure S22. Eigenvector index showing slowest modes of simulated trajectories.** **38**

**Figure S23. Time dependent Root Mean Square Deviation (RMSD) of DNA heavy atoms for the 4 simulated trajectories.** **39**

**Figure S24. Per-residue Root Mean Square Fluctuations10 of the DNA nanostructures from the four simulated trajectories.** **40**

**Figure S25. Boxplot of RMSF[10] showing the values for Component A and nanopore A•B in solution and interacting with membranes.** **41**

**Figure S26. Top-down images showing representative structures for the four simulated trajectories, coloured by per-residue RMSF[10].** **41**

**Table S8. Calculated Cy3-Cy5 distances as an approximation for the average diameter of the nanopore, A•B, in each condition.** **42**

**Table S9. Area per lipid (APL) values for the two membrane simulations.** **42**

**Figure S27. Average angle of the lipid head groups relative to the bilayer normal.** **43**

**Figure S28. Evolution of the simulated per residue distance between the lipid bilayer and DNA nanopore, A•B, and component A.** **43**

**Figure S29. Analysis of different clustering cut-off values for the clustered lumen analysis of the membrane embedded DNA nanopore, A•B.** **44**

**Figure S30. A•B concentration dependent SRB release from SUVs.** **45**

**Figure S31. Characterization of Fura-2-encapsulated vesicles for the $Ca^{2+}$ influx assay.** **46**

## 1. Experimental

### 1.1. Materials

Unmodified, fluorophore-labeled, and cholesterol-modified DNA oligonucleotides were purchased from Integrated DNA Technologies on a 100 nmol scale with HPLC purification. 1,2-dioleoyl-*sn*-glycero-3-phosphocholine (DOPC), 1,2-dioleoyl-*sn*-glycero-3-phosphoethanolamine (DOPE), 1-palmitoyl-2-oleoyl-*sn*-glycero-3-phosphocholine (POPC) and 1,2-diphytanoyl-*sn*-glycero-3-phosphocholine (DPhPC) were procured from Avanti Polar Lipids (US). All other reagents and solvents were purchased from Merck (UK) unless specified.

### 1.2. DNA assembly

Equimolar mixtures of DNA oligonucleotides (1 µL each, stock concentration of 100 µM)(Table S2 for composition of DNA pores and components) were dissolved at 1 µM in a buffer solution of either buffer A (1x PBS; 137 mM NaCl, 2.7 mM KCl, 10 mM $Na_2HPO_4$, 1.8 mM $KH_2PO_4$, pH 7.4), buffer B (300 mM KCl, 15 mM Tris-HCl, pH 7.4) or buffer C (12 mM $MgCl_2$ in 0.6x TAE (40 mM Tris, 20 mM acetic acid), pH 7.4) to a final volume of 100 µL. Folding was achieved on a BioRad T100 Thermocycler (UK) using a program involving heating to 95 °C and holding for 0.5 min, then cooling to 75°C within 5 min, holding for 1 min before cooling to 4 °C at a rate of 0.5 °C per 1 min. Samples were stored at 4 °C for up to 1 week.

### 1.3. PAGE

The assembled DNA nanostructure and component DNA oligonucleotides were analyzed with commercial 10% polyacrylamide gels (BioRad, UK) in 1x TBE buffer (100 mM Tris, 90 mM boric acid, 1 mM EDTA, pH 8.3). For gel loading, a solution of the DNA nanopores (2 µL, 1 µM) was mixed with folding buffer (13 µL, 2 mM $MgCl_2$ in 0.6x TAE, pH 7.4) and 6x gel loading dye (5 µL, New England Biolabs, UK). Gels were run at 115 V for 90 min at 4 °C. The gel bands were visualized by staining with ethidium bromide and UV illumination. A 100 bp marker (New England Biolabs, UK) was used as a reference standard.

### 1.4. Agarose gel electrophoresis

The assembled DNA nanostructures and component DNA oligonucleotides were analyzed with 2-3% agarose (Invitrogen, UK) gels in 1x TAE buffer (40 mM Tris, 20 mM acetic acid, 1 mM EDTA, pH 8.3). For gel loading, a solution of the DNA nanostructure (2 µL, 1 µM) was mixed with folding buffer (13 µL) and 6x gel loading dye (5 µL, New England Biolabs, UK). The gel was run at 60 V for 60 min at 4 °C. The gel bands were visualized by staining with ethidium bromide and UV illumination. A 100 bp marker (New England Biolabs, UK) was used as a reference standard.

### 1.5. Preparation of small unilamellar vesicles (SUVs)

DPhPC (100 µL, 10 mM) or POPC (100 µL, 10 mM) in chloroform was added to a 5 mL round bottom flask. The solvent was removed using a rotary evaporator (Buchi, Newmarket, UK) to yield a thin film, which was further dried under high vacuum (Buchi, Newmarket, UK) for 1 h. The lipid was re-suspended in 1 mL of either buffer A or buffer B. The solution was sonicated for 20 min at 30 °C and then equilibrated for 1 h before being extruded 25 times through a 0.1 µm polycarbonate membrane (Avanti Polar Lipids, US) using the extruder kit (Avanti Polar Lipids, US). SUVs were then stored at 4 ˚C and used within 48 h.

### 1.6. Melting temperature ($T_m$) analysis using UV-vis spectroscopy

UV melting profiles were obtained using a 10 mm quartz cuvette (Hellma Analytics, Southend-on-Sea, UK) in a Varian Cary 300 Bio UV–vis spectrophotometer (Agilent, Cheadle, UK) equipped with a Peltier element (Agilent, Cheadle, UK). Samples were analyzed at 200 nM and SUVs composed of DPhPC at 200 µM lipid concentration. Samples were analyzed by monitoring the change in absorbance at 260 nm as the temperature was increased from 20 to 80 °C at a rate of 1 °C/min. Melting profiles were then background corrected, and the 1$^{st}$ derivative calculated to identify the $T_m$.

### 1.7. Electrophoretic mobility shift assay

For binding titrations, component $A^{\Delta C}$ or A (5 µL, 1 µM) was mixed with component $B^{\Delta C}$ or B (1 µM stock) in buffer A yielding concentrations of 0 to 0.5 µM in a final volume of 20 µL. In the case of A-SUV vs $B^{\Delta C}$ or B, component A (5 µL, 1 µM) was first added to SUVs (5 µL, 100 nm, 16.7 nM). After incubation for 30 min at 30 °C, 6x gel loading dye (5 µL, New England Biolabs, Hitchin, UK) was added, the samples were mixed and loaded onto a thermally equilibrated 2-3% agarose gel. The gel was run in 1x TAE buffer, pH 8.3 at 60 V for 60 min at 4 °C. Staining and molecular markers were as described in section 1.4. Band intensities were analyzed using ImageJ and normalized as $(1-(I_A-I_{background}))$. The normalized intensities were then fit to a Langmuir curve to determine the $K_d$.

Kinetic assembly titrations, component $A^{\Delta C}$ (5 µL, 1 µM) was mixed with $B^{\Delta C}$ (5 µL, 1 µM) in buffer A to a final volume of 20 µL. Samples were incubated at 30 °C for 0, 1, 5, 10, 15, 20, 25 and 30 min while shaking at 500 rpm. Samples were prepared in reverse time order and after all samples were prepared, samples were crashed in ice to arrest pore formation. For assembly locked components $A^{\Delta C}L^A$ vs $B^{\Delta C}L^B$ the keys, $K^A$ and $K^B$ (1 µL, pre-mixed, 5 µM) were also added to each timepoint. Samples were mixed with 6x gel loading dye (5 µL) and then loaded onto thermally equilibrated 10% PAGE. The gel was run in 1x TBE buffer at 115 V for 90 min at 4 °C. Staining and molecular markers were as described in section 1.3.

### 1.8. FRET assay on pore assembly

For binding titrations, the assembly of A•B was investigated using a fluorescence spectrophotometer (Cary Eclipse, Agilent, Cheadle, UK). To a plastic Eppendorf tube was added $A^{\Delta C}$, A or $AL^A$ (12 µL, 1 µM), $B^{\Delta C}$, B or $BL^B$ (0 µL, 1.2 µL, 2.4 µL, 6 µL, 12 µL, 24 µL; 1 µM), SUVs (0 µL, 6 µL; 1 mM lipid, 7.22 nM SUV) and buffer B to a final volume of 120 µL. The tube was then incubated at 30 °C for 30 min while shaking at 750 rpm. The combined solution was then added to a 10 mm quartz cuvette (Hellma Analytics, Southend-on-Sea, UK),

which was placed in the fluorescence spectrophotometer and scanned ($ex_{545\ nm}$, $em_{555\text{-}725\ nm}$). Pre-folded A•B was used as a control for maximum assembly. Where SUVs were used, A or $AL^{A}$ and SUVs were mixed and left to bind for 10 min prior to addition of $B^{\Delta C}$, B or $BL^{B}$. The emission intensity of the donor (Cy3) were normalized between $A^{\Delta C}$ or A and a pre-folded control pore $(A•B)^{\Delta C}$, $(A•B)^{1C}$, or A•B. A 1:2 of A:B was used as an internal control. Due to the ability of the donor (Cy3) to donate to multiple acceptor (Cy5) molecules, this was set to the same binding level as the pre-assembled control and was used as an anchor point for $K_d$ determination.

For kinetic assembly, the assembly of A•B was investigated by monitoring Cy3 emission ($ex_{550nm}$, $em_{570nm}$) using a fluorescence spectrophotometer (Cary Eclipse, Agilent, Cheadle, UK). To a 10 mm quartz cuvette (Hellma Analytics, Southend-on-Sea, UK), $A^{\Delta C}$ or A (2.5 µL, 1 µM) was added to SUVs (0 µL, 1.25 µL; 1 mM lipid, 7.22 nM SUV) and buffer B (97.5, 96.25 µL) and the signal left to stabilize for 5 min. Then, $B^{\Delta C}$ or B (50 µL, 1 µM) was rapidly added and mixed. Pore formation was monitored for 1 h. Where SUVs were used, A and SUVs were mixed and left to bind for 10 min prior to the start of the run.

FRET Efficiency (E) calculations were achieved using the equation (2):

$$E = 1 - \frac{I_{DA}}{I_D} \qquad (2)$$

Where $I_{DA}$ is the donor intensity in the presence of the donor and acceptor; $I_D$ is the donor intensity in the absence of the acceptor.

The inter-fluorophore (Cy3-Cy5) distance was calculated using equation (3):

$$E = \frac{1}{1 - (\frac{r}{R_0})^6} \qquad (3)$$

*E* stands for FRET efficiency, *r* is the donor-acceptor separation distance, $R_0$ is the Förster distance where E = 50%.

### 1.9. Dual-color FCCS analysis to measure the $K_d$ of pore assembly

Dual-color fluorescence cross-correlation measurements were carried out on a commercial laser scanning microscope (ConfoCor 3, Carl Zeiss, Jena, Germany) equipped with a 40x water immersion objective. $A^{\Delta C}$ and $B^{\Delta C}$ were labeled with the spectrally non-overlapping fluorophores Alexa488 and Alexa647, respectively. Cross-correlation measurements were performed using a 635 nm secondary dichroic mirror with a 505-540 nm bandpass filter in the green channel, and a 650 nm longpass filter in the red channel. Laser power was adjusted such that the brightness ratio of Alexa647 to Alexa488 was roughly 3:1.

At a 1:1 binding stoichiometry, the bound fraction of either ${}^{Alexa488}A^{\Delta C}$ ($X_g$) or ${}^{Alexa647}B^{\Delta C}$ ($X_r$) can be calculated from the number of double labelled particles ($N^{rg}$) relative to the total number of each particle ($N^g$ for ${}^{Alexa488}A^{\Delta C}$ or $N^r$ for ${}^{Alexa647}B^{\Delta C}$) using equations 4 and 5:

$$X_g = \frac{N^{rg}}{N^g} \qquad (4)$$

$$X_r = \frac{N^{rg}}{N^r} \quad (5)$$

The number of particles of ${}^{Alexa488}A^{\Delta C}$ or ${}^{Alexa647}B^{\Delta C}$ detected in the green or red channel, respectively, was obtained from the fit of the respective autocorrelation (for $N^g$ and $N^r$) and cross-correlation (for $N^{rg}$) function $G(\tau)$ by the one-component model for 2D translational diffusion shown in equation 6:[1]

$$G(\tau) = 1 + \frac{1}{N(1 + \tau/\tau_D)} \quad (6)$$

where $\tau_D$ represents the diffusion time through the confocal volume. The fraction of bound particles, $X_g$ and $X_r$, was corrected by accounting for the difference in size of the green and red detection volumes, $V_g$ and $V_r$, by using the following formulas:[2]

$$X_{g/v} = X_g * \frac{V_g}{V_{eff}} \quad (7)$$

$$X_{r/v} = X_r * \frac{V_r}{V_{eff}} \quad (8)$$

where $V_{eff}$ represents the effective cross-correlation volume, which is defined by the following equation:[3]

$$V_{eff} = \pi^{3/2} * \left(\frac{\omega_r^2 + \omega_g^2}{2}\right)^{\frac{3}{2}} * S \quad (9)$$

where $S$ is a structural parameter, which was set equal for both channels, $S_g = S_r = 6$, and $\omega$ is the respective lateral radius of the confocal volume, $\omega_g$ or $\omega_r$, of the green or red confocal volume. Confocal volumes, $V$, in the red and green channels were calculated as:[1]

$$V = \pi^{3/2} * \omega^3 * S \quad (10)$$

The lateral radii, $\omega_g$ and $\omega_r$, we obtained from calibration experiments measuring labels with known diffusion coefficients *(D)*, Rhodamine 6G (Merck, Germany) and Alexa647 maleimide (Jena Bioscience, Germany), using the equation:[1]

$$\omega^2 = 4D\tau_D \quad (11)$$

On each day that data were collected, the maximum achievable cross-correlation was determined using a DNA-duplex carrying both an Alexa488 and Alexa647 fluorophore (IDT, USA) to represent 100% binding.

For binding measurements, ${}^{Alexa488}A^{\Delta C}$ was mixed with ${}^{Alexa647}B^{\Delta C}$ in buffer B, to a final volume of 60 µL. In other measurements, the concentration of ${}^{Alexa647}B^{\Delta C}$ was varied from 0.5 nM to 300 nM, while the concentration of ${}^{Alexa488}A^{\Delta C}$ (7.5 µL, 100 nM) remained constant. The samples were then incubated at 30 °C for 30 min with shaking. After incubation, each ratio mixture was measured separately. Measurements where ${}^{Alexa647}B^{\Delta C}$ remained constant (9.6 µL, 100 nM), while the concentration of ${}^{Alexa488}A^{\Delta C}$ was varied.

To determine $K_d$, the resulting volume-corrected fractions of bound particles $X_{g/v}$ and $X_{r/v}$ were fitted to a Langmuir isotherm:

$$X = A * \frac{C}{C + K_d} \quad (12)$$

where $C$ represents concentration of the varied component.

By design, a dc-FCCS experiment shows the saturation value below 100%, and a slight overestimation of the cross-correlation is observed in the red channel (Figure S8a). The former originates from the non-complete overlapping of the confocal volumes for the red and green lasers. The latter stems from the bleed-through of the green-labelled particles into the red channel at higher ratios of green-to-red particles, which is pronounced at ratios higher than 10:1.[4] To minimize the effect, we performed before every FCCS experiment a control measurement using a cross-correlation standard. This is a fluorescently double labelled DNA-duplex. Within 5% accuracy, the control measurements showed the same deviation from 100% saturation level, and the same ratio of saturation levels between the red and the green channels.

### 1.10. Preparation of giant unilamellar vesicles (GUVs)

A solution of POPC lipids (5 µL, 10 mM in chloroform) was added to an indium tin-oxide (ITO) coated glass slide. Within 5 min the solvent evaporated, and a dried lipid film was formed. The glass slide was then inserted in a vesicle prep device (Nanion Technologies, Munich, Germany). An O-ring was added around the patch. Sucrose (300 µL, 1 M in water) was added to the lipid film patches confined by the O-ring. Finally, another ITO glass slide was applied from the top, resulting in a sealed chamber. An alternating electric field was applied between the two slides by using a voltage program of 3 V, 5 Hz for 120 min. The solution was collected and stored at 4 °C.

### 1.11. Confocal laser scanning microscopy

A GUV suspension (10 µL, 130 µM lipid concentration) was added to a FluoroDish (World Precision Instruments, Hitchin, UK) with buffer (500 µL, 1x TAE, 500 mM NaCl, pH 8.1). The solution gently mixed. After adding component A (10 µL, 1 µM) to the dish, the solution was mixed thoroughly and left for 10 min to ensure membrane binding. Component $B^{\Delta C}$ (10 µL, 1 µM) was then added following by mixing of the solution. The mixture was left for 5 min to allow the GUVs sink to the bottom of the dish. The FluorDish (World Precision Instruments, Hitchin, UK) was placed under the microscope and GUVs were located by visualization using a 96x optical zoom. The sample was then viewed through the brightfield and at 570 nm for $^{Cy3}$A and 670 nm for $^{Cy5}$B and images were acquired.

### 1.12. Preparation of planar lipid bilayers on a glass slide and smFRET and single particle tracking

Planar lipid bilayers were formed on glow discharged glass slides provided by Oxford NanoImaging (Oxford, UK). SUVs composed of DPhPC in buffer A (15 µL, 1 mM) were placed onto the support and left for 15 min. Some solution (~ 5 µL) was then supplanted with $H_2O$

and left for 1-2 min. This was repeated 3x. After the 3rd wash with $H_2O$, the solution was washed with buffer A. Slides were used within 1 h and topped up with buffer A as necessary. smFRET and single particle tracking was performed using a NanoImager S (Oxford NanoImaging, Oxford, UK) by Jon Shewring from Oxford NanoImaging. Structures were added (1 µL, 1 nM in buffer A) to planar lipid bilayers composed of DPhPC on glass slides.

### 1.13. Enzyme digestion assay

A solution of the lipid DPhPC (100 µL, 10 mM) in chloroform was added to a 5 mL round bottom flask. The solvent was removed using a rotary evaporator (Buchi, Newmarket, UK) to yield a thin film. The lipid was re-suspended in buffer B (1 mL), sonicated for 20 min at 30 °C and then equilibrated for 3 h. $(A•B)^{\Delta C}$, $(A•B)^{1C}$ or A•B (2 µL, 1 µM), nuclease-free water (58 µL) and 2x Bal-31 buffer (60 µL; 40 mM Tris-HCl, 1.2 M NaCl, 24 mM $MgCl_2$, 24 mM $CaCl_2$, 2 mM EDTA) were added to a 10 mm quartz cuvette (Hellma Analytics, Southend-on-Sea, UK). For assays with vesicles, $(A•B)^{\Delta C}$, $(A•B)^{1C}$ or A•B (2 µL, 1 µM) were first incubated with LUVs (18 µL, 1 mM DPhPC) for 1 h then added to the 10 mm quartz cuvette with nuclease-free water (40 µL) and 2x Bal-31 buffer (60 µL, New England Bioscience, Hitchin, U.K.). Fluorescence was monitored using a Varian Cary Eclipse fluorescence spectrophotometer (Agilent, Cheadle, UK) at 570 nm and excited at 555 nm. After 5 min, Bal-31 (0.75 µL, New England Bioscience, Hitchin, U.K.) was added, and the fluorescence emission was monitored for 15 min.

### 1.14. Circular dichroism

Solution-phase circular dichroism (CD) spectroscopy was performed on a Jasco-810 spectropolarimeter (Kromatec Ltd, Great Dunmow, UK). A micro-volume quartz Couette flow cell with ~0.5 mm annular gap and quartz capillaries were used (Kromatec Ltd, Great Dunmow, UK). CD spectra were acquired for DNA nanopores (1.4 µM) between 320-190 nm.

### 1.15. Linear dichroism

Solution-phase flow linear dichroism (LD) spectroscopy was performed on a Jasco-810 spectropolarimeter (Kromatec Ltd, Great Dunmow, UK) using a photo elastic modulator 1/2 wave plate. A micro-volume quartz Couette flow cell with ~0.5 mm annular gap and quartz capillaries were used (all from Kromatec Ltd, Great Dunmow, UK). Molecular alignment was achieved by applying the constant flow of the sample solution between two coaxial cylinders, a stationary quartz rod and a rotating cylindrical capillary. LD spectra were acquired with laminar flow obtained by maintaining the rotation speed at 3000 rpm and processed by subtracting non-rotating baseline spectra. DNA nanopores were assayed at 1.4 µM and SUVs composed of POPC at 500 µM lipid concentration.

### 1.16. Simulation preparation

DNA nanopore A•B, and component A were recreated in caDNAno, then converted to all atom models in python.[5] The poly-thymine linker regions at the pore termini were then constructed using the MolSoft ICM software suite.[6] TEG-Cholesterol lipid anchors were parametrized using cgenff[7] and attached using pyMol.[8] CHARMM36 compatible topology files were then

generated using psfgen.[9] Initial structures of A•B and A were minimized in a vacuum for 10,000 steps (2 fs), then simulated for 100,000 steps (2 ns) using an elastic restraint network[10] derived from the ENRG webserver.[11]

DNA nanopore A•B and component A were simulated in 1 M KCl and TIP3 water prepared in VMD.[12] Nanopore A•B was simulated in 13 x 11 x 15 nm box totaling 437k atoms and component A was simulated in a box of 16 x 14 x 19 nm totaling 6.5k atoms. A 1 ns *NpT* equilibration was run to equilibrate box size and pressure before a 50 ns *NvT* equilibration to further relax the DNA nanostructures. Production simulations were then run in in the *NpT* ensemble.

For simulations of membrane tethered component A and membrane-inserted A•B, VMD was used to generated membranes and orient the DNA nanostructures while maintaining favorable cholesterol oritentations.[13] The orientation of each structure was informed by experimental data derived from linear dichroism. The membrane-spanning nanopore A•B was simulated in a 12 x 12 x 12 nm box of 1 M KCl, bisected by a bilayer composed of POPC lipids for a total of 141k atoms. The membrane-tethered component A was simulated in a 15 x 15 x 16 nm box in the same conditions totaling 303k atoms. While the fixed atom restraints were placed on all atoms except those of the lipid tails, which were then thermally equilibrated over 0.5 ns of dynamics in the *NvT* ensemble as the temperature was increased to 301 K.[14] Fixed atom restraints were replaced with harmonic restraints, with a spring constant of 1 kcal/mol/Å$^2$, on the heavy atoms of the DNA phosphate backbones. Simulation box size and pressure were equilibrated in the *NpT* ensemble for 3.5 ns, with harmonic restraints being lowered by 0.5 kcal/mol/Å$^2$ every 0.5 ns. Unrestrained dynamics in the *NvT* ensemble allowed the system to fully equilibrate, and production simulations were performed in the *NpT* ensemble.

Production simulations were performed at 301K and 1.013 bar pressure, maintained with the Langevin thermostat[15] and the Nosé-Hoover Langevin piston method.[16] Simulations were performed in NAMD,[17] a smooth switching algorithm[18] with a switch distance of 8 Å, a cut off of 10 Å and a pair list distance of 12 Å was implemented for van der Waals interactions. A 2 fs time step was used and hydrogen bond lengths were constrained using the SETTLE and SHAKE algorithms.[19] Particle Mesh Ewald electrostatics were computed over a cubic grid with a 1.0 Å spacing[20] and periodic boundary conditions.[21] Equilibration simulations were performed on a on a single GPU 1080Ti workstation and production runs were performed in parallel on 850 CPU cores of the UCL Grace HPC facility.

### 1.17. Simulation analysis

Analysis was performed using GROMACs[22] and VMD tools on the production simulations, after discarding the initial 10 ns, graphs were prepared using *ggplot*[23] and *RStudio.*[24]

#### 1.17.1. RMSF[10]

gmx_covar and gmx_aneig were used to investigate the ten top quasi-harmonic modes[25] of root mean squared fluctuations (RMSF) of the DNA backbone heavy atoms, averaged per-residue, to interrogate structural dynamics of the DNA nanostructures while accounting for thermal noise and stochastic motion.

### 1.17.2. Clustering

gmx_cluster was used to prepare snapshots of the membrane spanning A•B DNA nanostructure trajectory. Clustering was performed with a cut-off of 0.35 nm using the gromos method.[26]

### 1.17.3. Lumen analysis

Clustered coordinates were analyzed using HOLE,[27] with a channel-end radius of 0.8 nm and a sampling distance of 0.25 nm. To account for asymmetry of the DNA nanostructure, coordinates were then rotated and analyzed again.

### 1.17.4. Lipid analysis

gmx_gangle was used to measure the angle of phosphate and nitrogen atoms in the lipid head groups, split by lipid leaflet, compared to the bilayer normal, over the initial equilibration simulations. Production simulations were analyzed using gmx_rms, and the VMD plugins density_profile_too[28] and MEMBPLUGIM[29] to determine lipid RMSF, average lipid density and area-per-lipid, respectively.

### 1.18. Nanopore current recordings

Single-channel current measurements were achieved using an integrated chip-based, parallel bilayer recording setup (Orbit Mini, Nanion Technologies, Munich, Germany) with multielectrode-cavity-array (MECA) chips (IONERA, Freiburg, Germany). Bilayers were formed from DPhPC (10 mg/mL in octane). The electrolyte solution was 1 M KCl and 10 mM HEPES, pH 7.4. To achieve pore insertions, a 2:1 mixture of nanopore A•B and 0.5% OPOE (*n*-octyloligooxyethylene, in 1 M KCl, 10 mM HEPES, pH 7.4) was added to the *cis* side of the bilayer. Successful insertion was observed by detecting current steps. For triggered assembly, membranes were preincubated with $AL^A$ and $BL^B$ and 0.5% (v/v) OPOE. After 10 minutes, key strands were added and successful insertions were observed by detecting current steps. The current traces were not Bessel-filtered and were acquired at 10 kHz using Element Data Recorder software (Element s.r.l., Cesena, Italy). Single-channel analysis was performed using Clampfit software (Molecular Devices, Sunnyvale, CA, USA).

### 1.19. Preparation of fluorophore-filled SUVs and dye release assay

A solution of DPhPC lipids (100 µL, 10 mM) in chloroform was added to a 5 mL round bottom flask. The solvent was removed using a rotary evaporator (Buchi, Newmarket, UK) to yield a thin film, which was further dried under high vacuum (Buchi, Newmarket, UK) for 1 h. The lipid was re-suspended in buffer A containing the fluorophore sulforhodamine B (SRB, 50 mM), sonicated for 20 min at 30 °C and then equilibrated for 3 h at 4° C. SUVs were then extruded 25 times through a 100 nm polycarbonate membrane (Avanti Polar Lipids, US) using an extruder kit (Avanti Polar Lipids, US). The non-encapsulated SRB was removed using a NAP-25 column (Cytivia, UK) and SUVs were exchanged into buffer D (0.2 M KCl, 10 mM Tris pH 7.4). Purified SUVs were used within 48 h and gently resuspended immediately prior to use.

For the release assays, A•B was folded at 1 µM in buffer C using the 15 h folding protocol while components A and B were folded at 2 µM. The SUV suspension with encapsulated SRB (10 µL) and buffer D (110 µL, 80 µL) were added to a 10 mm quartz cuvette (Hellma Analytics, Southend-on-Sea, UK). Fluorescence was monitored using a Varian Cary Eclipse fluorescence spectrophotometer (Agilent, Cheadle, UK) at 586 nm and excited at 565 nm. After 5 min, A, A+B, A•B, $AL^A$+$BL^B$ or $AL^A$+$BL^B$+$K^A$+$K^B$ (30 µL, or 60 µL; 1 µM in buffer C) was added to a final volume of 150 µL. After 55 min of monitoring fluorescence, samples were mixed with a 1% (v/v) solution of Triton X-100 (10 µL) to lyse all vesicles to identify maximum SRB release. Maximum fluorescence emission and the fluorescence prior to addition of A•B (or components) were used to calculate the extent of release as %.

### 1.20. Preparation of Fura-2-filled SUVs and $Ca^{2+}$ influx assay

A solution of the lipid POPC (100 µL, 10 mM) in chloroform was added to a 5 mL round bottom flask. The solvent was removed using a rotary evaporator (Buchi, Newmarket, UK) to yield a thin film, which was further dried under high vacuum (Buchi, Newmarket, UK) for 1 h. The lipid was re-suspended in buffer E (500 mM NaCl, 100 mM HEPES, pH 7.4) containing the fluorophore Fura-2 (100 µM). The solution was sonicated for 20 min at 30 °C and then equilibrated for 3 h. SUVs were extruded 25 times through a 100 nm polycarbonate membrane (Avanti Polar Lipids, US) using an extruder kit (Avanti Polar Lipids, US). The non-encapsulated dye was removed using Illustra MicroSpin S-400 spin columns (Cytivia, UK). SUVs were then subjected to dynamic light scattering with a Malvern Zetasizer Nano S (Malvern Pananalytical, Malvern, UK) to confirm the vesicles' diameter. Purified SUVs were used within 48 h and gently resuspended immediately prior to use. For $Ca^{2+}$ influx assays, the SUV suspension with encapsulated Fura-2 (30 µL) and buffer E (138.3 µL, 145.8 µL, 148.33 µL) were added to a 10 mm quartz cuvette (Hellma Analytics, Southend-on-Sea, UK Fluorescence was monitored using a Varian Cary Eclipse fluorescence spectrophotometer (Agilent, UK) at 510 nm and excited at 340 and 380 nm. After 2.5 min, $CaCl_2$ (16.7 µL, 3 mM in $H_2O$) was added and allowed to stabilize for a further 2.5 min. At 5 min, $(A•B)^{4C}$ (15 µL, 7.5 µL, 5 µL, 1 µM in buffer B) was added to a final volume of 200 µL. After 30 min of monitoring fluorescence, samples were mixed with a 1% solution (v/v) of Triton X-100 (10 µL) to lyse all vesicles to identify maximum $Ca^{2+}$ influx. $Ca^{2+}$ influx was monitored as the ratio of the change in emission at each excitation wavelength as a ratio of 340/380 nm. The maximum 340/380 nm ratio following addition of Triton X-100 was used to normalize all traces.

## 2. Tables and Figures

**Table S1. Names, modifications and sequences of DNA oligonucleotides used for folding A, B, A•B and variants.**

| ID | Sequence 5′ → 3′ |
|---|---|
| $A_1$ | ATTAGCGAACGTGGATTTTGTCCGACATCGGCAAGCTCACTTTTTCGACT |
| $A_2$ | TTGCCGATGTCGGACTTTTGAACGATCTTCGCCTGCTGCGTTTTGTGAGC |
| $B_1$ | AGGCGAAGATCGTTCTTTTCCTGCACGTCCAACTGGTCAGTTTTCGCAGC |
| $B_2$ | AGTTGGACGTGCAGGTTTTTCCACGTTCGCTAATAGTCGATTTTCTGACC |
| $L^A$ | GGACCATGGTTCCACGTTCGCTAATAGTCGA |
| $L^B$ | TGGTCAAGCAGAACGATCTTCGCCTGCTGCG |
| $K^A$ | TCGACTATTAGCGAACGTGGAACCATGGTCC |
| $K^B$ | CGCAGCAGGCGAAGATCGTTCTAGCTTGACC |
| $A_1$ (chol) | Sequence of $A_1$ carrying a cholesterol *via* tri(ethylene glycol) TEG linker at the 3′ terminus |
| $A_2$ (chol) | Sequence of $A_2$ carrying a cholesterol *via* a TEG linker at the 3′ terminus |
| $B_1$ (chol) | Sequence of $B_1$ carrying a cholesterol *via* a TEG linker at the 3′ terminus |
| $B_2$ (chol) | Sequence of $B_2$ carrying a cholesterol *via* a TEG linker at the 3′ terminus |
| $^{Cy3}A_1$ | $^{Cy3}$ATTAGCGAACGTGGATTTTGTCCGACATCGGCAAGCTCACTTTTTCGAC T |
| $^{Cy5}B_1$ | $^{Cy5}$AGGCGAAGATCGTTCTTTTCCTGCACGTCCAACTGGTCAGTTTTCGCA GC |
| $^{Alexa488}A_1$ | $^{Alexa488}$ATTAGCGAACGTGGATTTTGTCCGACATCGGCAAGCTCACTTTTTC GACT |
| $^{Alexa647}B_1$ | $^{Alexa647}$AGGCGAAGATCGTTCTTTTCCTGCACGTCCAACTGGTCAGTTTTCG CAGC |

$^{Cy3}$X = Cy3 fluorophore; $^{Cy5}$X = Cy5 fluorophore; $^{Alexa488}$X = Alexa488 fluorophore; $^{Alexa647}$X = Alexa647 fluorophore

**Table S2. Names and strand compositions of structures used for the DNA nanopore with biomimetic triggered assembly**

| | Composition |
|---|---|
| $A^{\Delta C}$ | $A_1$, $A_2$ |
| $B^{\Delta C}$ | $B_1$, $B_2$ |
| A | $A_1$, $A_2$ (chol) |
| B | $B_1$, $B_2$ (chol) |
| $^{Cy3}A^{\Delta C}$ | $^{Cy3}A_1$, $A_2$ |
| $^{Alexa488}A^{\Delta C}$ | $^{Alexa488}A_1$, $A_2$ |
| $^{Cy3}A$ | $^{Cy3}A_1$, $A_2$ (chol) |
| $^{Cy5}B^{\Delta C}$ | $^{Cy5}B_1$, $B_2$ |
| $^{Alexa647}B^{\Delta C}$ | $^{Alexa647}B_1$, $B_2$ |
| $^{Cy5}B$ | $^{Cy5}B_1$, $B_2$ (chol) |
| $A^{\Delta C}L^{A}$ | $A_1$, $A_2$, $L^A$ |
| $B^{\Delta C}L^{B}$ | $B_1$, $B_2$, $L^B$ |
| $AL^A$ | $A_1$, $A_2$ (chol), $L^A$ |
| $BL^B$ | $B_1$, $B_2$ (chol), $L^B$ |
| $(A{\bullet}B)^{\Delta C}$ | $A_1$, $A_2$, $B_1$, $B_2$ |
| $(A{\bullet}B)^{1C}$ | $A_1$, $A_2$ (chol), $B_1$, $B_2$ |
| A•B | $A_1$, $A_2$ (chol), $B_1$, $B_2$ (chol) |
| $(A{\bullet}B)^{4C}$ | $A_1$ (chol), $A_2$ (chol), $B_1$ (chol), $B_2$ (chol) |

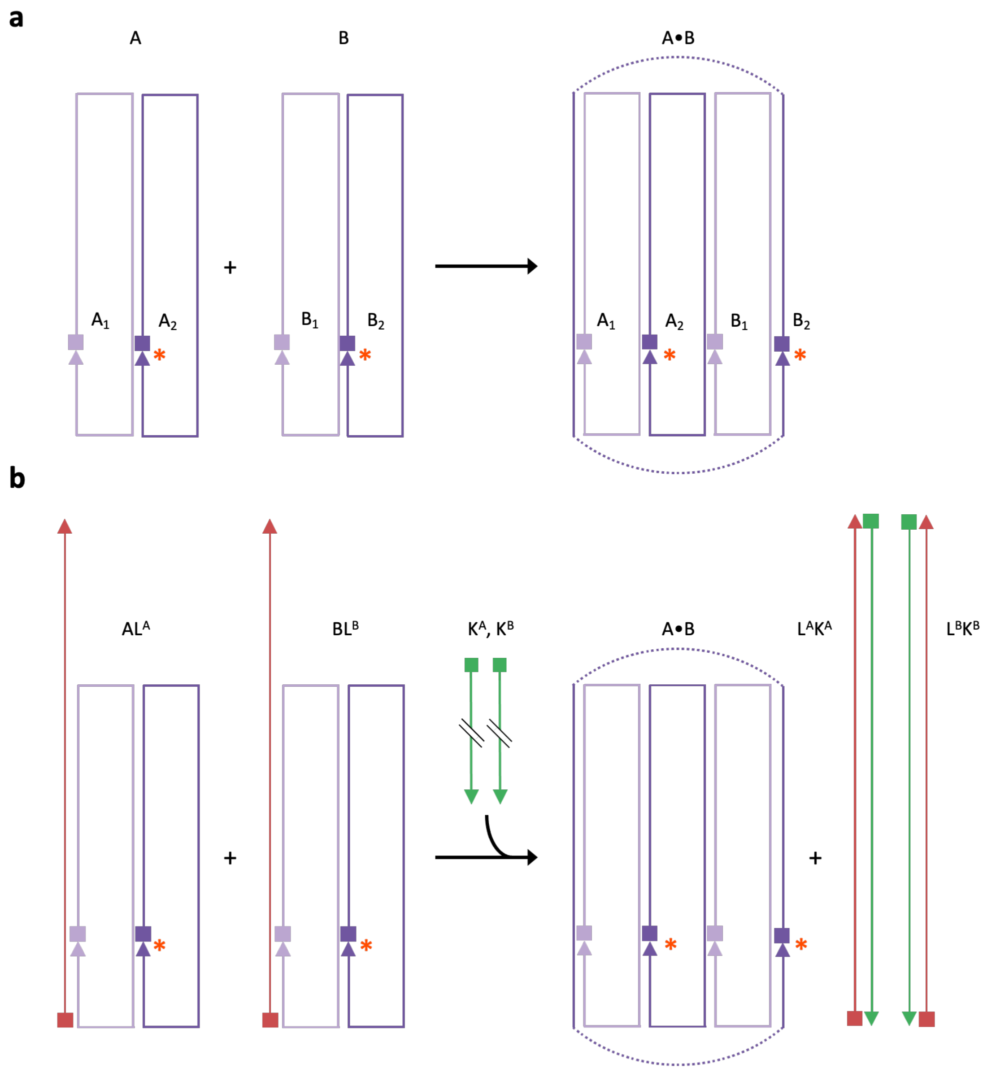


**Figure S1. 2D strand maps of direct and triggered assembly of A•B.** (a) 2D strand map schematic the components A and B and assembly into the 4HB nanopore, A•B. (b) 2D strand map schematic of the assembly locked components $AL^A$ and $BL^B$ indicating the mechanism of assembly into A•B upon addition of the keys ($K^A$ and $K^B$, green) to remove the locks ($L^A$ and $L^B$, red). The resultant lock-key duplexes are indicated at the right. Squares represent the 5' and triangles the 3' end of DNA, orange asterisks represent cholesterol modifications attached to the 3' end of the indicated strand *via* a tri(ethylene)glycol (TEG) linker. Strands that make up the pore are shown in alternating blue and purple. The crossovers at the top and bottom of each strand consist of four thymine bases each.

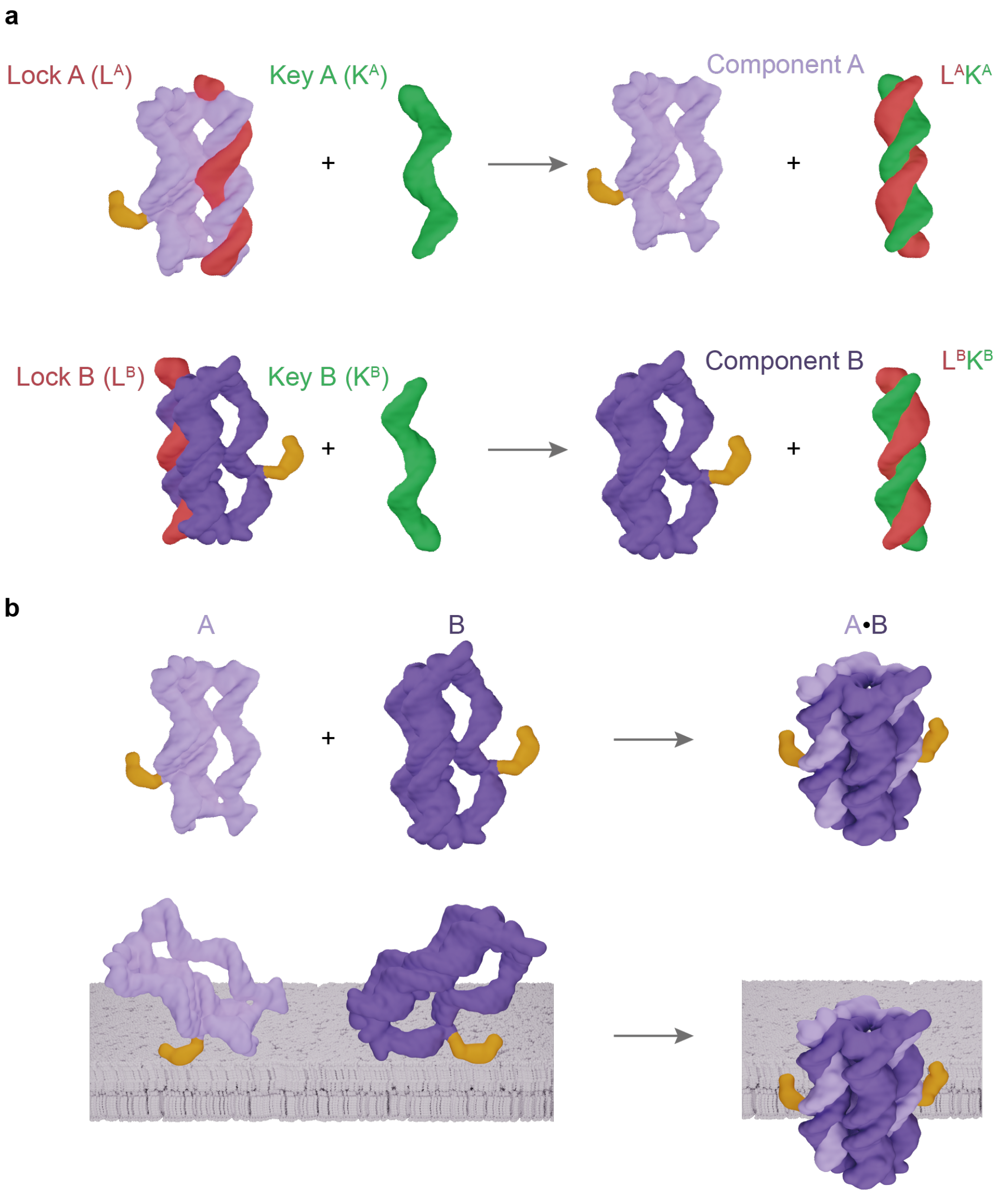


**Figure S2. Schematic illustrating the triggering mechanism and assembly in solution and on membranes.** (a) Assembly locked pre-pore components ($AL^A$ and $BL^B$) are composed of pore components (A and B) plus a lock strands ($L^A$ and $L^B$), respectively, which prevent pore formation. Addition of key strands ($K^A$ and $K^B$) unzip each lock strand *via* a toehold mechanism leaving the unlocked components (A and B). (b) A and B are free to self-assemble in solution (top) or on the surface of the membrane (bottom) to form active ion channels.

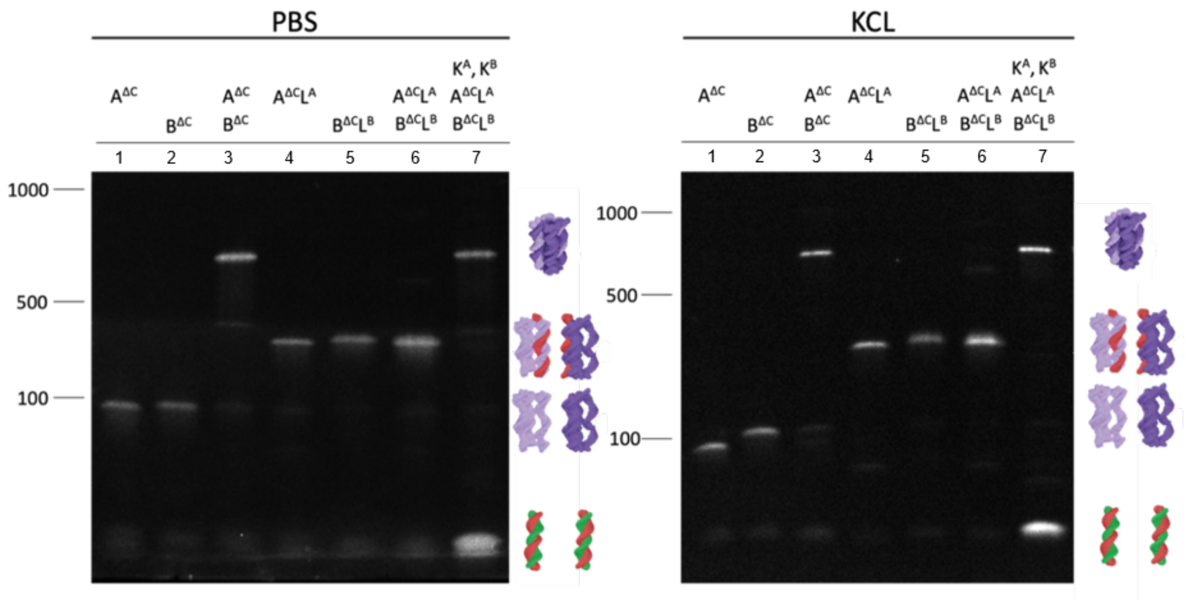


**Figure S3. PAGE characterization of A•B pore formation.** Confirmation of the assembly of the nanopore $(A{\bullet}B)^{\Delta C}$ from component parts in PBS (left) and KCl (300 mM KCl, 15 mM Tris-HCl, pH 7.4; right). Assembly of $(A{\bullet}B)^{\Delta C}$ from unlocked components and the efficacy of the locking mechanism to control and trigger pore assembly was assessed by native PAGE. Addition of $A^{\Delta C}$ and $B^{\Delta C}$ in a 1:1 stoichiometric ratio results in the assembly of $(A{\bullet}B)^{\Delta C}$ (Lanes 1-3). Addition of $A^{\Delta C}L^{A}$ + $B^{\Delta C}L^{B}$ in a 1:1 stoichiometric ratio show no interaction and remain in their constituent parts (Lanes 4-6). However, addition of keys $K^{A}$ and $K^{B}$ in a 1:1 stoichiometric ratio removes the locks and results in the formation of $(A{\bullet}B)^{\Delta C}$ (Lane 7). Addition of keys in a 1:1 stoichiometric ratio is also sufficient for complete removal of the corresponding lock (Lane 7). Samples were incubated at 30 °C for 30 min before being loaded onto the gel. 10% (left) and 12% (right) native PAGE were run in 1x TBE buffer at 90 V for 115 min at 4 °C.

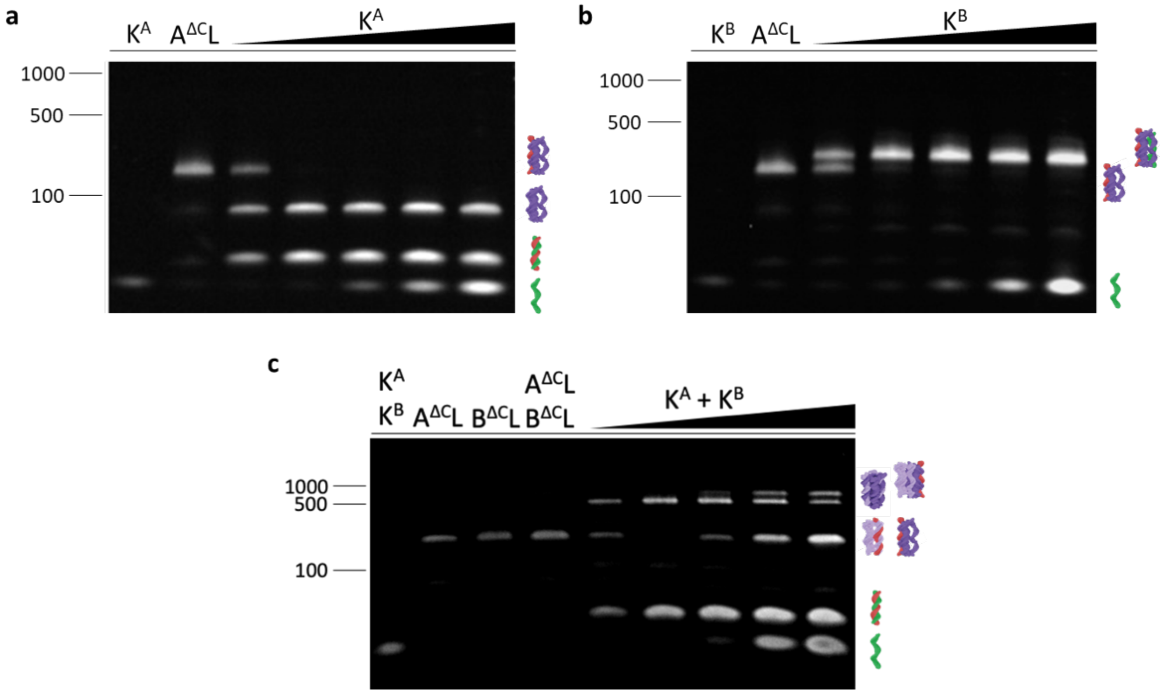


**Figure S4. Gel electrophoretic mobility shift assay on the key specificity and the concentration dependence of triggered pore assembly.** (a) Key $K^A$ was mixed with assembly-locked component $A^{\Delta C}L^A$ at molar ratios ranging from 1:0.5 to 1:10 of $A^{\Delta C}L{:}K^A$. The gel indicates that $A^{\Delta C}L^A$ is quantitatively converted to $A^{\Delta C}$ at a stoichiometric ratio of 1:1. Excess key does not interact with $A^{\Delta C}$. (b) Key $K^B$ was added to assembly locked component $A^{\Delta C}L^A$ at molar ratios ranging from 1:0.5 to 1:10 $A^{\Delta C}L{:}K^B$. The gel indicates that $A^{\Delta C}L^A$ is not unlocked and instead forms a new structure $A^{\Delta C}L^AK^B$ at a stoichiometric ratio of 1:1 and that excess key does not interact further once a stoichiometric structure has been formed. (c) Keys, $K^A$ and $K^B$ were mixed with the assembly locked components $A^{\Delta C}L^A$ and $B^{\Delta C}L^B$ in molar ratios of 1:0.5 to 1:10 of $A^{\Delta C}L^A$, $B^{\Delta C}L^B$: $K^A$, $K^B$. The gel indicates that when all four elements are at a 1:1:1:1 stoichiometric ratio that the pore $(A{\bullet}B)^{\Delta C}$ and the two duplexes $K^AL^A$ and $K^BL^B$ are the sole products. As the amount of keys increases, other products such as the assembly - incomplete $A^{\Delta C}B^{\Delta C}D$ emerge, where D = $L^A$, $L^B$, $K^A$, or $K^B$. At a 1:1:10:10 stoichiometric ratio the keys inhibit pore formation resulting in a significant amount of assembly-locked $A^{\Delta C}L^A$ and $B^{\Delta C}L^B$, free keys and minor amounts of product $A^{\Delta C}B^{\Delta C}D$ as well as the assembled $(A{\bullet}B)^{\Delta C}$.

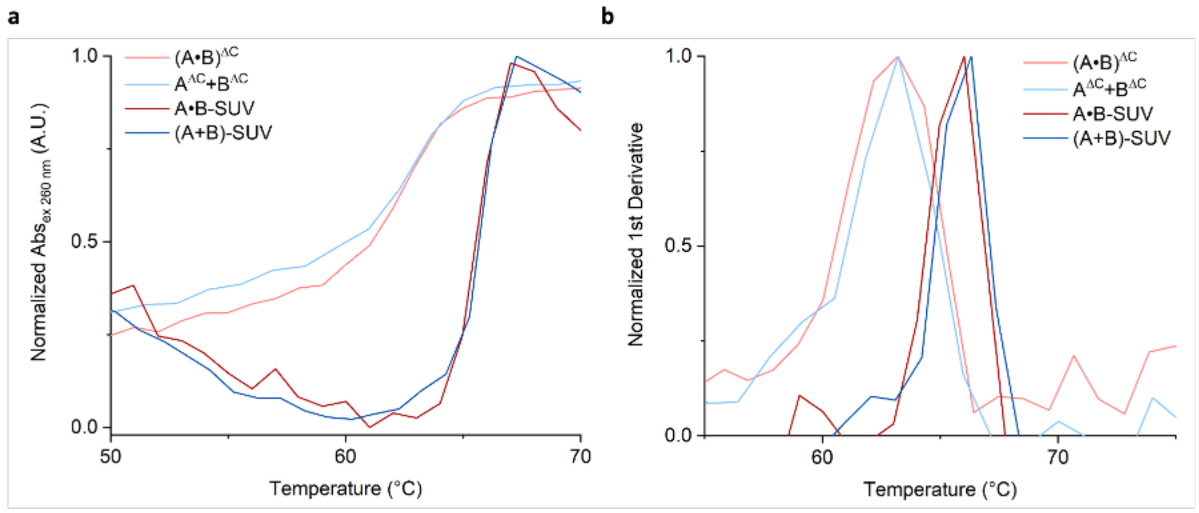


**Figure S5. UV-melting profiles.** (a) UV-melting profiles of nanopore A•B when pre-annealed (light blue) or directly assembled from A and B (light red). The melting profiles were assessed in solution or when inserted into small unilamellar vesicles (SUVs) composed of DPhPC lipids with a diameter of 170 nm. A•B was assessed at 0.2 µM with 200 µM DPhPC lipid in 150 µL of PBS buffer. Samples were melted using a temperature ramp of 1 °C/min from 20 and 80 °C. Each trace represents an average from three independent repeats. (b) 1$^{st}$ derivative of the melting profiles used to identify the melting temperature ($T_m$) of each structure.

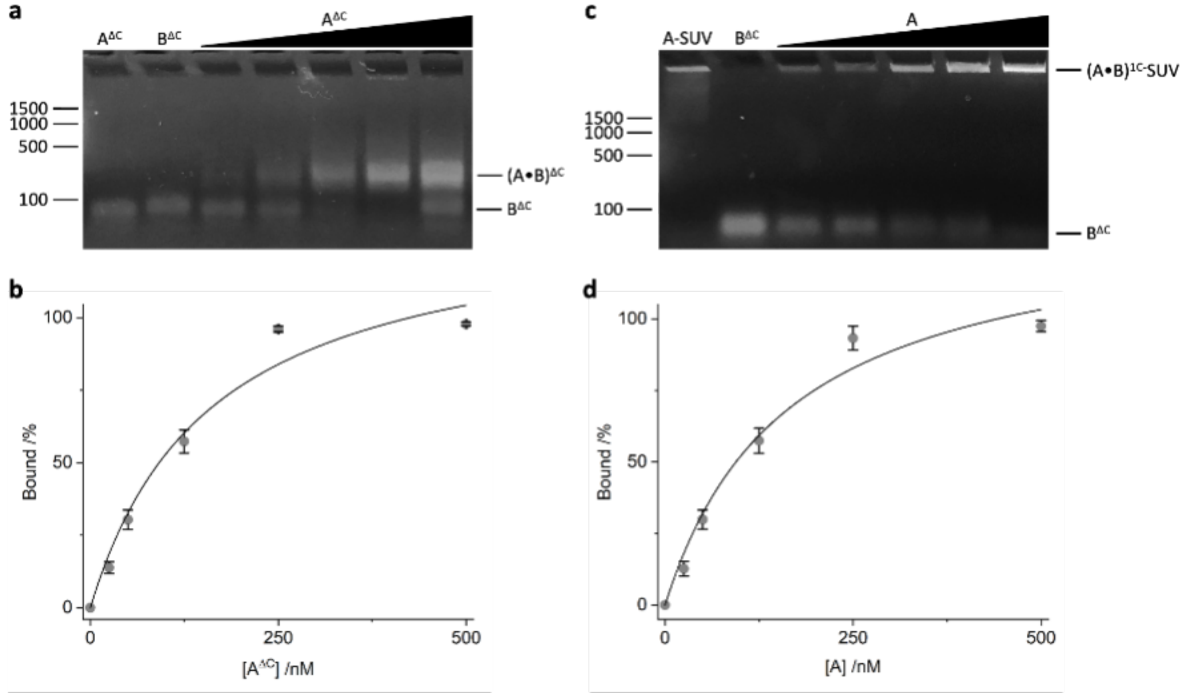


**Figure S6. Gel electrophoretic mobility shift assay on the formation of the A•B pore.** (a) A representative 3% agarose gel demonstrating the binding of $A^{\Delta C}$ and $B^{\Delta C}$ in solution. Addition of increasing concentrations of $A^{\Delta C}$ results in the formation of the $(A•B)^{\Delta C}$ nanopore complex, which is larger than the individual components and migrates through the gel more slowly. (b) Binding curve of $(A•B)^{\Delta C}$ complexation fit to a Langmuir curve revealed a $K_d$ of 1.54 ± 0.23 x $10^{-7}$ M. The data represent averages and standard deviations from three independent experiments. (c) A representative 2% agarose gel demonstrating binding of A-SUV and $B^{\Delta C}$ on the membrane surface. Addition of increasing concentrations of A results in the formation of the $(A•B)^{1C}$ membrane-bound complex, which is unable to migrate into the gel matrix and remains in the well. (d) Binding curve of $(A•B)^{1C}$ complexation. Fitting to a Langmuir curve revealed a $K_d$ of 1.64 ± 0.14 x $10^{-7}$ M. The data represent averages and standard deviations from three independent experiments. All gels were run in 1x TAE buffer at 60 V for 60 min. The major markers of a 100 bp DNA ladder are indicated on the left of each gel. Bands are identified at the top and right.

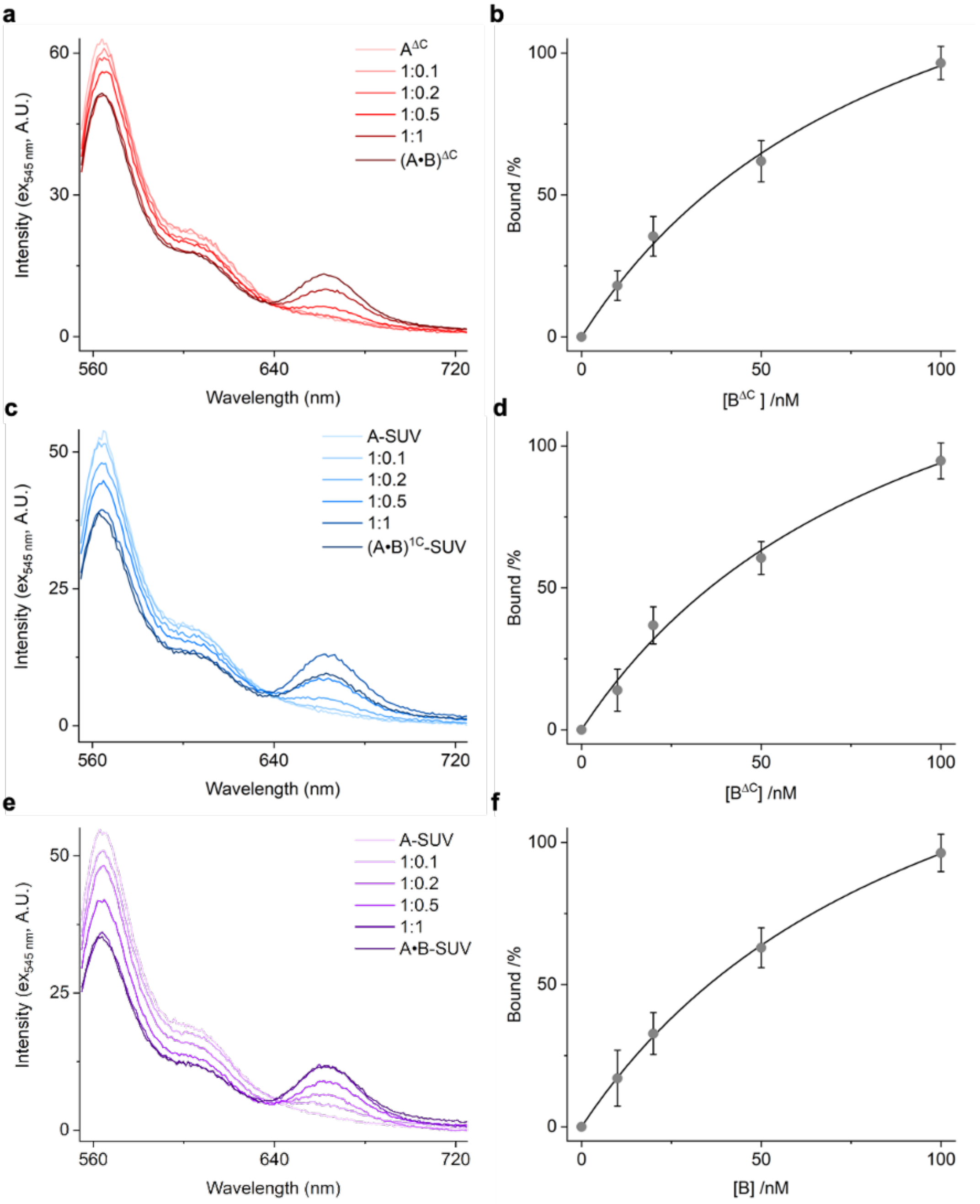


**Figure S7. Förster resonance energy transfer (FRET) analysis on pore assembly.** Components A and B carry the FRET pair Cy3 and Cy5, respectively. Increasing concentrations of $^{Cy5}$B (FRET acceptor) are added to $^{Cy3}$A (FRET donor) resulting in assembly into $^{Cy3}$A•$^{Cy5}$B leading to close proximity and FRET, which is manifest as a decrease in Cy3 emission intensity and an increase in Cy5 emission intensity. (a) Pore assembly titration of $A^{\Delta C}$ vs $B^{\Delta C}$ in solution. Ratios are of $A^{\Delta C}$:$B^{\Delta C}$ (b) Binding curve of $(A•B)^{\Delta C}$ pore assembly based on (a) displayed as percent bound. (c) Pore assembly titration of the membrane tethered A-SUV vs $B^{\Delta C}$. Ratios are of A:$B^{\Delta C}$ (d) Binding curve of $(A•B)^{1C}$ pore assembly based on (c) displayed as percent bound. (e) Pore assembly titration of A vs B on the membrane surface. Ratios are of A:B (f) Binding curves of A•B pore assembly based on (e) displayed as percent bound. The data displayed in (b, d, and f) represent averages and standard deviations from three independent experiments.

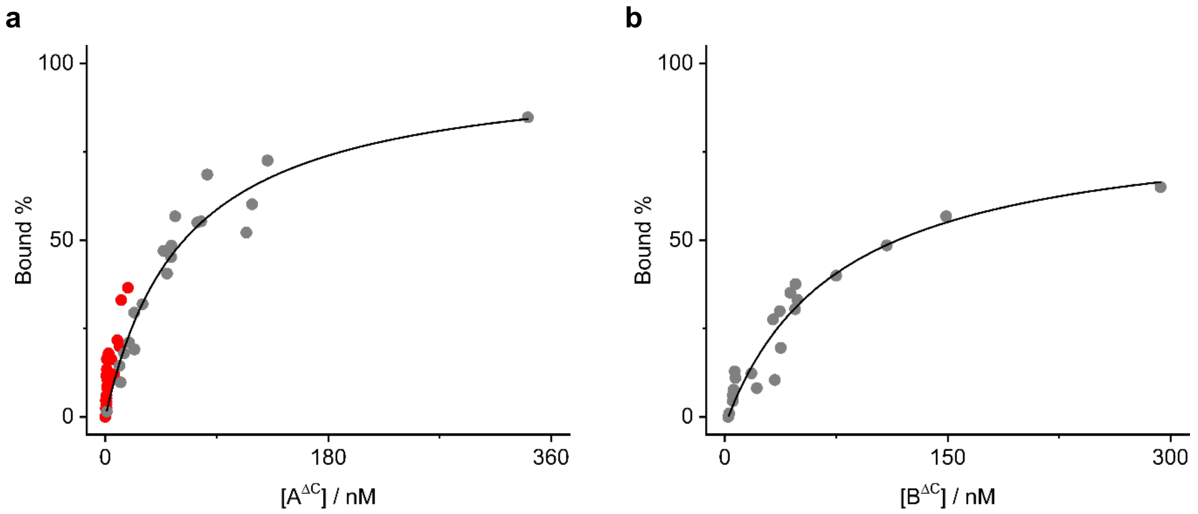


**Figure S8. Dual-color fluorescence cross-correlation spectroscopy analysis of pore assembly.** Components $A^{\Delta C}$ and $B^{\Delta C}$ carried an Alexa488 and an Alexa647 fluorophore, respectively. (a) Example binding curve of $(A{\bullet}B)^{\Delta C}$ pore formation as a function of $^{Alexa488}A^{\Delta C}$ concentration (grey dots). The concentration of $^{Alexa647}B^{\Delta C}$ was held constant at 16 nM. Each point represents a separate sample measurement. The solid line represents a Langmuir isotherm that was fitted to the data, yielding a $K_d$ = 6,39 ± 1,07 x10$^{-8}$ M. The average of both repeats yielded a $K_d$ of 7.17 ± 0.77 x 10$^{-8}$ M with maximum observed binding of A = 101%. The red dots represent the same experiment, but with $^{Alexa647}B^{\Delta C}$ held constant at 1 nM. (b). Example binding curve of $(A{\bullet}B)^{\Delta C}$ pore formation as a function of $^{Alexa647}B^{\Delta C}$ concentration (grey dots). The concentration of $^{Alexa488}A^{\Delta C}$ was held constant at 15 nM. The solid line represents a Langmuir isotherm that was fitted to the data, yielding a $K_d$ = 7.99 ± 1.68 x10$^{-8}$ M. The average of three repeats resulted in a $K_d$ of 6.22 ± 1.25 x 10$^{-8}$ M and maximum observed binding of B = 84%.

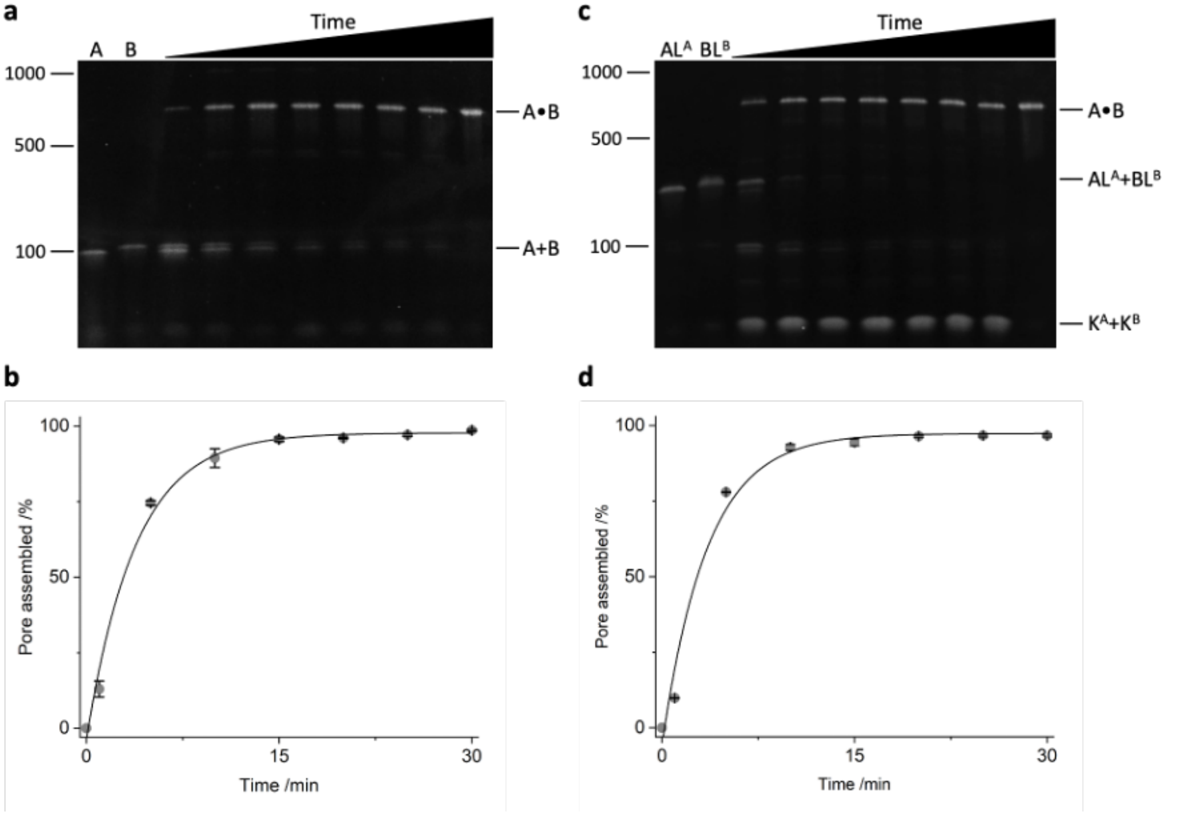


**Figure S9. PAGE kinetic electrophoretic mobility shift assays of direct and triggered pore assembly.** (a) PAGE titration to assess A•B formation from the direct assembly of $A^{\Delta C}$+$B^{\Delta C}$. (b) Quantitative analysis of the kinetic data from (a) *via* plotting the normalized amount of assembled A•B against time. The amount of assembled A•B was extracted from the gel band brightness as 1-($I_A$-$I_{background}$) where I = intensity. (c) PAGE titration to assess A•B assembly from the triggered assembly of $AL^A$+$BL^B$+$K^A$+$K^B$. (d) Quantitative analysis of the kinetic data from (a) *via* plotting the normalized amount of assembled A•B against time. The amount of assembled A•B was extracted from the gel band brightness as 1-($I_{AL}$-$I_{background}$) where *I* = intensity. 12% native PAGE run in 1x TBE buffer at 115 V for 90 min at 4 °C. Time points are 1, 5, 10, 15, 20, 25, and 30 min. Each trace represents data from three independent repeats. The major markers of a 100 bp DNA ladder are indicated at the left.

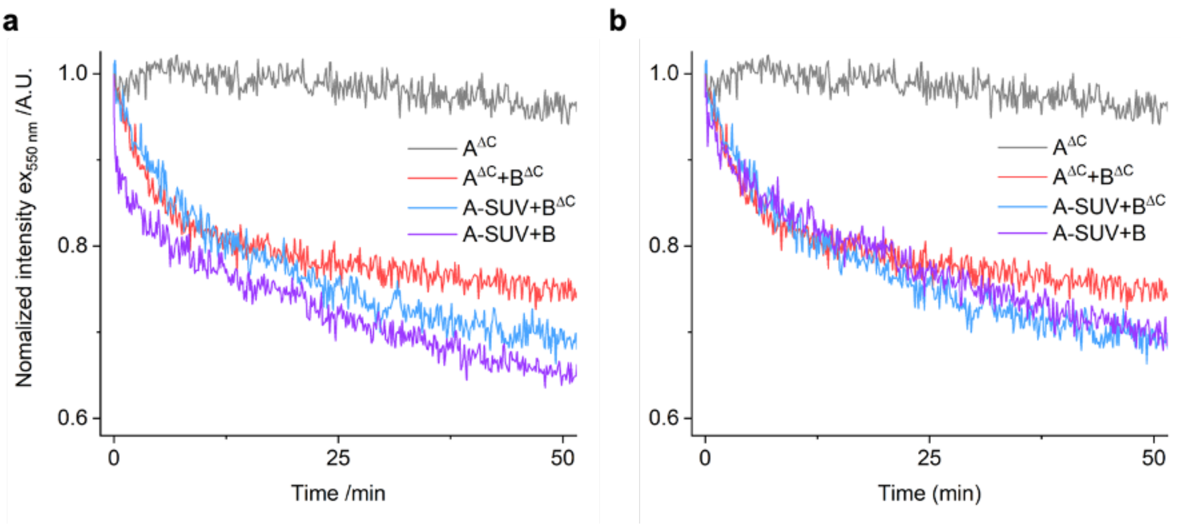


**Figure S10. Pore assembly kinetics monitored via FRET.** (a) Kinetic fluorescence analysis of pore assembly over time in solution ($A^{\Delta C}$+$B^{\Delta C}$, red), when one component is tethered to the membrane (A-SUV+$B^{\Delta C}$, blue), and on the membrane surface (A-SUV+B, purple). A control trace (grey) was also added using $A^{\Delta C}$ and buffer only. (b) The same as (a), but the initial rapid decline of A-SUV+B (purple) has been removed. Each trace is an average of three independent repeats.

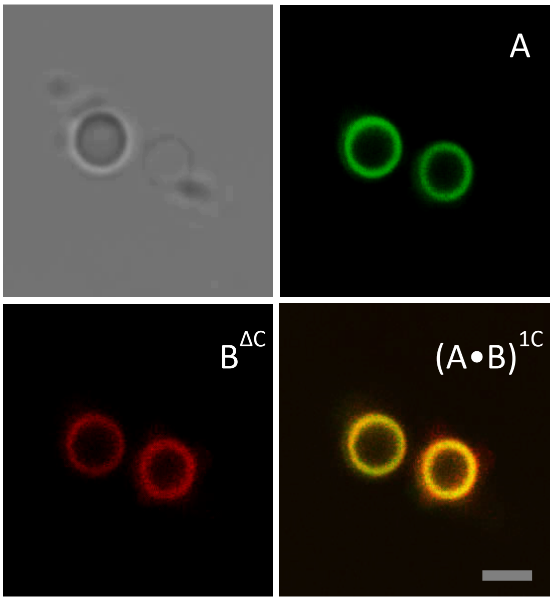


**Figure S11. Confocal microscopy images confirm cholesterol-mediated membrane tethering of (A•B)1C to GUVs and pore assembly on the membrane surface.** Component A was modified with a Cy3 fluorophore and B with a Cy5 fluorophore. Images were obtained using a 96x oil objective, scale bar 5 µm. (Top left) Brightfield image of two GUVs composed of POPC lipids. (Top right) Green channel shows a ring around the GUVs demonstrating the $^{Cy3}$A tethering to the membrane. (Bottom left) Red channel shows $^{Cy5}B^{\Delta C}$, which cannot interact with the membrane on its own, has bound to the membrane tethered component A. (Bottom right) Overlay of the red and green channels demonstrating $(A{\bullet}B)^{1C}$ formation and membrane tethering.

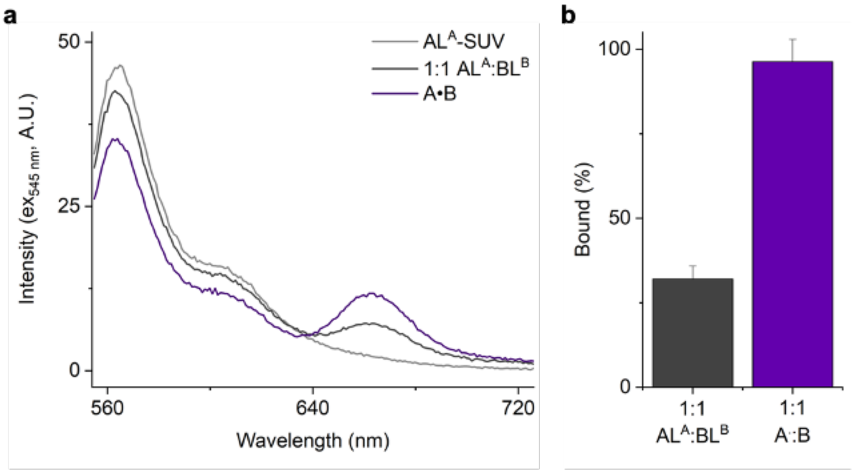


**Figure S12. FRET control between assembly-locked components at the membrane surface.** (a) Fluorescence spectra of the assembly locked components $AL^A$ and $BL^B$ to confirm pore assembly. The control also serves to assess the effect of Cy3-Cy5 proximity on the membrane surface. (b) Normalized bar graph comparing the FRET signal observed from a 1:1 stoichiometric ratio of assembly locked components, $AL^A$ and $BL^B$, on the membrane surface to FRET signal observed from the direct assembly of A+B in a 1:1 stoichiometric ratio. Each trace is an average of three independent repeats, the standard deviation shown as the error bars in the bar chart.

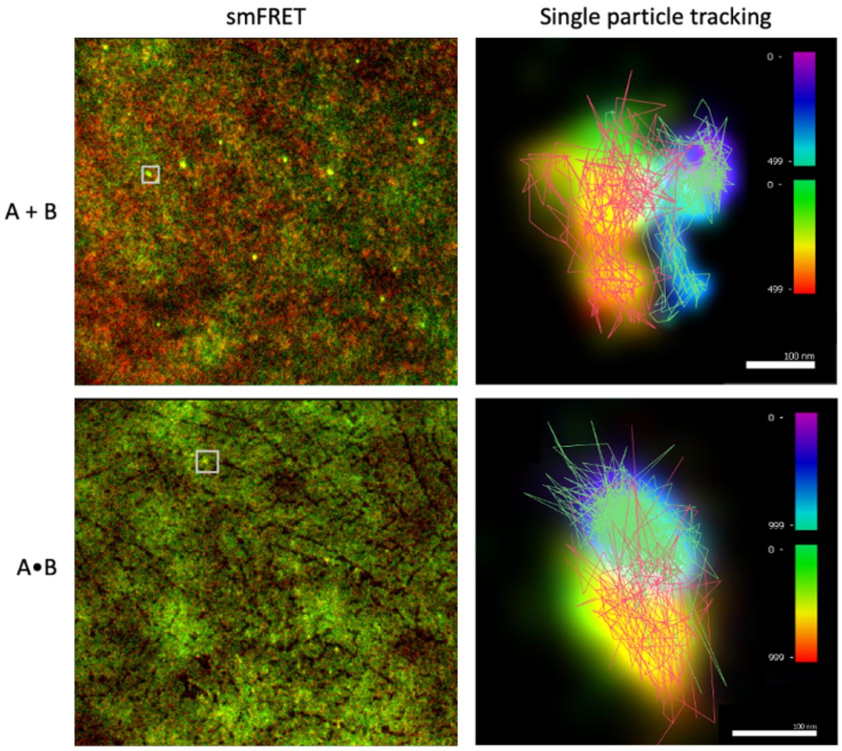


**Figure S13. smFRET and single particle tracking confirm A+B assembly at the membrane surface.** Single-molecule FRET (smFRET) and single particle tracking images confirm pore assembly from the monomer component A and B (top) in comparison to a control pre-mixed A•B (bottom). Bright green spots indicate the formation of A•B in the smFRET images (e.g., grey boxes). A carries a Cy3 fluorophore at the 5' end of strand $A_1$ and B carries a Cy5 fluorophore at the 5' end of strand $B_1$. Pore assembly from A and B was monitored on the supported lipid bilayer membrane at 62.5 pM. Single particle tracking experiments were monitored for 500 and 1000 s for A+B and pre-mixed A•B, respectively.

**Table S3. Summarized FRET efficiency (E) data for pore assembly derived from the FRET pore assembly binding titrations and pore assembly kinetics.** The pre-annealed control for each of the three conditions was derived from the FRET binding data and was used as a benchmark. Averages and standard deviations were calculated from at least three independent repeats.

| Condition | Pre-folded control (E) | Binding (E) | *In situ* yield: binding (%) | Kinetics (E) | *In situ* yield: kinetics (%) |
|---|---|---|---|---|---|
| $A^{\Delta C}$ + $B^{\Delta C}$ | 0.27 ± 0.07 | 0.26 ± 0.07 | 96.3 ± 42.1 | 0.26 ± 0.02 | 95.6 ± 31.1 |
| A-SUV + $B^{\Delta C}$ | 0.28 ± 0.02 | 0.26 ± 0.03 | 94.8 ± 14.7 | 0.31 ± 0.05 | 113 ± 22.7 |
| A-SUV + B | 0.36 ± 0.03 | 0.34 ± 0.03 | 96.3 ± 13.4 | [a] 0.35 ± 0.02<br>[b] 0.31 ± 0.04 | 98.7 ± 11.4<br>87.2 ± 15.4 |

[a] derived from kinetic trace with initial drop included
[b] derived from kinetic trace with initial drop removed

**Table S4. Names, modifications, and sequences of DNA oligonucleotides used for folding R, S and $S_{NP}$.**

| ID | **Sequence 5′ → 3′** |
|---|---|
| $R^{\Delta C}$ | TAGTCGATTTTATCCATGCA |
| R | Sequence of $R^{\Delta C}$ carrying a cholesterol *via* a tri(ethylene) glycol (TEG) linker at the 3′ terminus |
| $^{Cy3}R^{\Delta C}$ | $^{Cy3}$TAGTCGATTTTATCCATGCA |
| $^{Cy3}R$ | Sequence of $^{Cy3}R^{\Delta C}$ carrying a cholesterol *via* a TEG linker at the 3′ terminus |
| S | TGCATGGATAAAATCGACTA |
| $^{Cy5}S$ | TGCATGGATAAAATCGACTATTTTT$^{Cy5}$ |
| 1 | ACA GGA TTT TCG CCT GCT GGG GCA AAC CAG CGT GGA CCG CTT TTT TGG CTA TTC TTT TGA TTT ATA AGG GAT TTT GCC GAT TTC GGA A |
| 2 | CAA CTC TCT CAG GGC CAG GCG GTG AAG GGC AAT CAG CTG TTG TTT TCA ACA GCA TCC TGT TTC CGA AAT CGG CAT TAA AGA CCA GCT G |
| 3 | TCT CAC TGG TGA AAA GAA AAA CCA CCC TGG CGC CCA ATA CGC TTT TTC CCC GCG CGT TGG CCG ATT CAT TAA TGC AGC TGG CAC GAC A |
| 4 | GGC GAA ATG ATT GCT TTC ACC AGT GAG ATG TCG TGA CGT GGA TTT TTC CAC GTT CTT TAA TAG TGG ACT CTT GTT CCA AAC TGG AAC A |
| 5 | TGT TCC AAA TAG CCA AGC GGT CCA CGC TCC CTG AGG GGC GCC |
| 6 | CAT TAA TTT TTT CTC CTT CAC CGC CTG GGG TTT GCT TAT AAA |
| 7 | AGG GTG GGA ATC GGA CAA GAG TCC ACT AAA ATC CCC CCA GCA |
| 8 | TCA AAA GGT TTG GAC CAA CGC GCG GGG AGC GTA TTA GAG TTG |
| 5-ext | TGC ATG GAT AAA ATC GAC TAT TTT TGT TCC AAA TAG CCA AGC GGT CCA CGC TCC CTG AGG GGC GCC |
| $^{Cy5}$5-ext | TGCATGGATAAAATCGACTATTTTT$^{Cy5}$GTTCCAAATAGCCAAGCGGTCCAC GCTCCCTGAGGGGCGCC |

$X^{Cy3}$ = Cy3 fluorophore; $X^{Cy5}$ = Cy5 fluorophore

**Table S5. Names and strand compositions of structures used for the model system.**

| | Composition |
|---|---|
| NP | 1, 2, 3, 4, 5, 6, 7, 8 |
| $S_{NP}$ | 1, 2, 3, 4, 5-ext, 6, 7, 8 |
| $^{Cy5}S_{NP}$ | 1, 2, 3, 4, $^{Cy5}$5-ext, 6, 7, 8 |
| $R^{\Delta C}$•S | $R^{\Delta C}$, S |
| R•S | R, S |
| $R^{\Delta C}$•$S_{NP}$ | $R^{\Delta C}$, 1, 2, 3, 4, 5-ext, 6, 7, 8 |
| R•$S_{NP}$ | R, 1, 2, 3, 4, 5-ext, 6, 7, 8 |
| $^{Cy3}R^{\Delta C}$• $^{Cy5}S$ | $^{Cy3}R^{\Delta C}$, $^{Cy5}S$ |
| $^{Cy3}R$•$^{Cy5}S$ | $^{Cy3}R$, $^{Cy5}S$ |
| $^{Cy3}R^{\Delta C}$•$^{Cy5}S_{NP}$ | $^{Cy3}R^{\Delta C}$, 1, 2, 3, 4, $^{Cy5}$5-ext, 6, 7, 8 |
| $^{Cy3}R$•$^{Cy5}S_{NP}$ | $^{Cy3}R$, 1, 2, 3, 4, $^{Cy5}$5-ext, 6, 7, 8 |

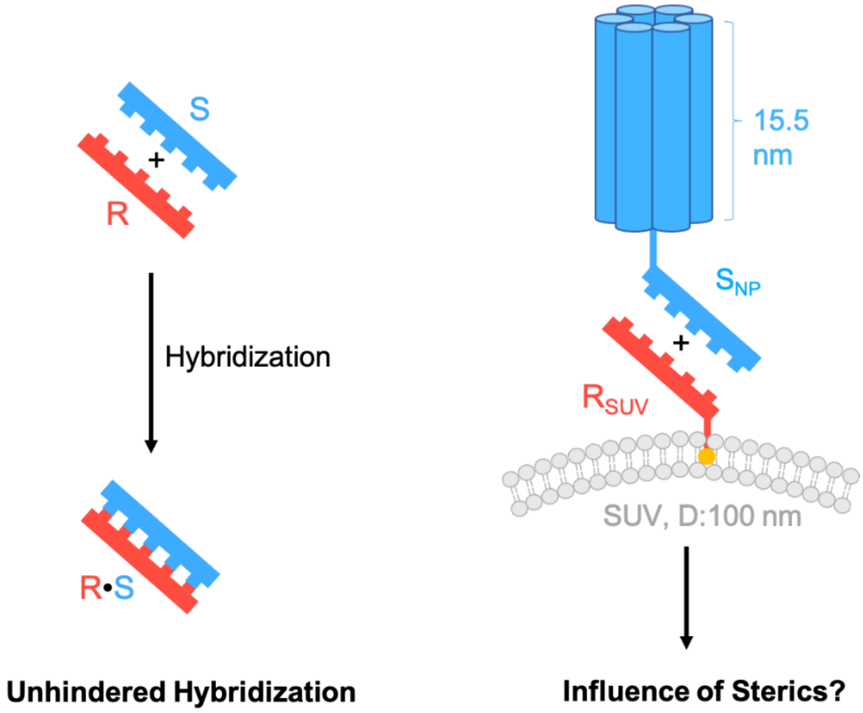


**Figure S14. Schematic illustration of the model system used to investigate the effect of sterics on DNA hybridization.** Schematic illustrating the model system used to probe the influence of sterics on DNA hybridization on membrane surfaces. The simplest of the four conditions, hybridization of the receptor (R, red) and the ligand, (S, blue) is shown on the left. The most sterically hindered of the four conditions, hybridization of the vesicle anchored receptor ($R_{SUV}$, red) and the ligand attached to the nanopore, ($S_{NP}$, blue) is shown on the right. The cholesterol lipid anchors are shown in orange inserted into a grey lipid bilayer. The SUVs used were composed of DOPC and DOPE in a 7:3 mole ratio and extruded to be ~100 nm in diameter. Only two of the four conditions are shown for clarity.

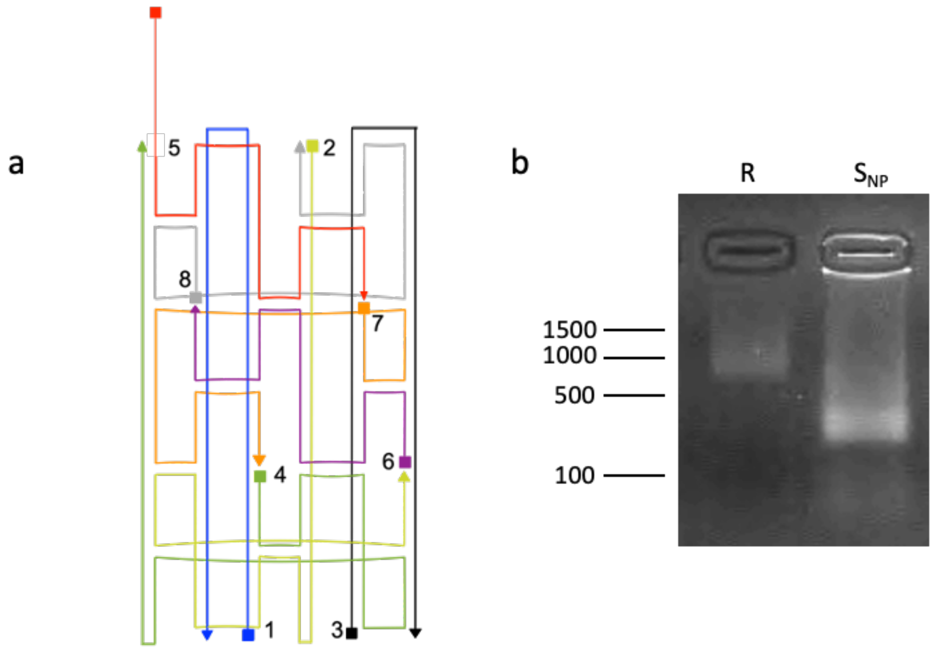


**Figure S15. Structure and assembly confirmation of $S_{NP}$.** (a) 2D strand map of $S_{NP}$. All strands are shown in unique colors and are numbered. Squares represent the 5' end of DNA and circles represent the 3' end of DNA. (b) 2% agarose gel confirming the presence of R and the assembly of $S_{NP}$.

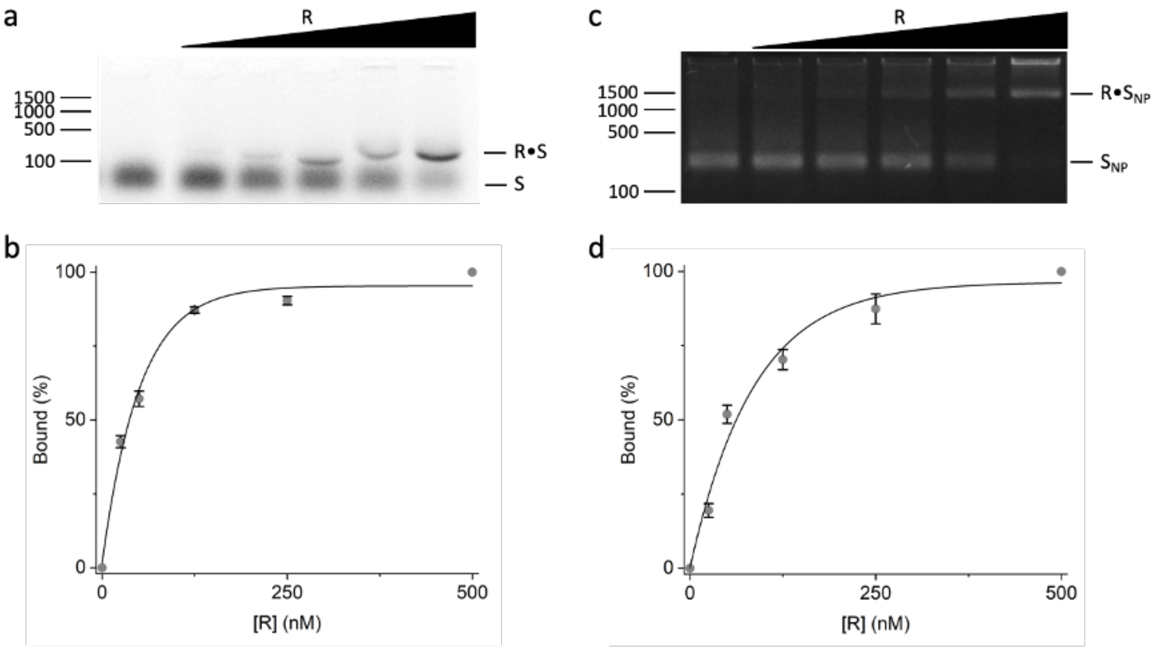


**Figure S16. Gel electrophoretic mobility shift assay to assess the binding of the receptor (R) to the ssDNA ligand (S) or nanopore-bound ligand ($S_{NP}$).** (a) A 3% agarose gel shift assay. Increasing concentrations of R were added to S resulting in the formation of the R•S dsDNA complex, which manifests as a more slowly migrating band. S carries a Cy5 fluorophore to enable imaging at low concentrations. (b) Binding curve of R•S complexation displayed as percent bound. The data were fit with a Langmuir binding curve and represent averages and standard deviations from 3 independent experiments. (c) A 2% agarose gel shift assay measuring the formation of the R•$S_{NP}$ complex. For improved separation, R modified with a cholesterol lipid anchor was sued. While the $S_{NP}$ is significantly more massive than R, the cholesterol modification interacts with the gel matrix resulting in significantly reduced R•$S_{NP}$ mobility. (d) Binding curve of R•$S_{NP}$ complexation displayed as percent bound. The data were fit with a Langmuir binding curve and represent averages and standard deviations from 3 independent experiments. All gels were run in 1x TAE buffer at 60 V for 60 min. The major markers of a 100 bp DNA ladder are indicated on the left.

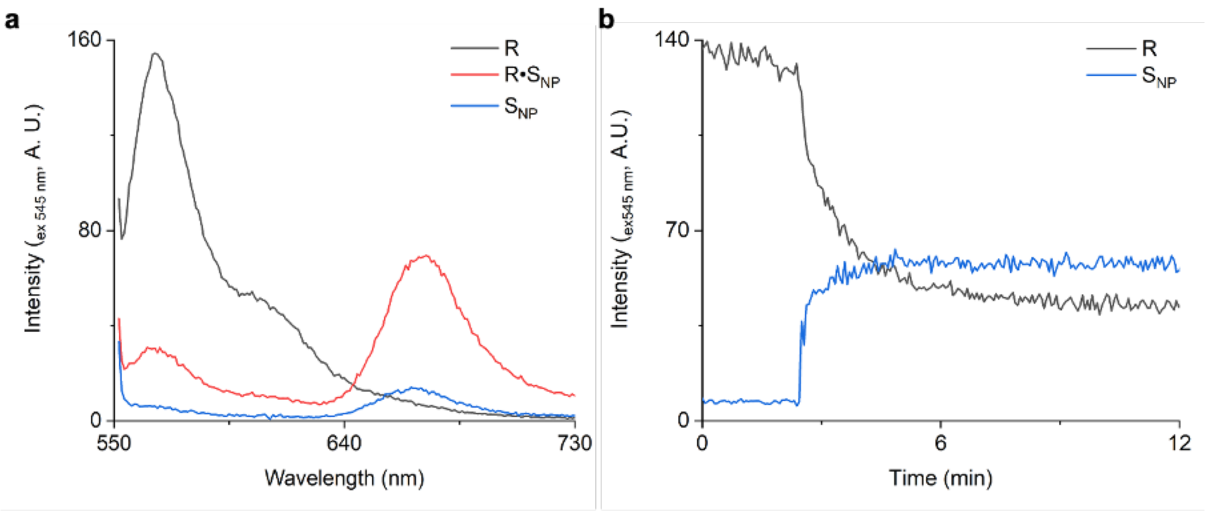


**Figure S17. Fluorescence analysis of the interaction of R and SNP labeled with the FRET pair, Cy3 and Cy5, respectively.** (a) Ensemble fluorescence measurement of the hybridization of R (black) and $S_{NP}$ (blue) resulting in complex R•$S_{NP}$ (red confirmed via FRET. (b) Kinetic fluorescence measurement of the change in intensity of $^{Cy3}$R and $^{Cy5}S_{NP}$ as a result of hybridization overtime. Measured in a 1:1 stoichiometric ratio of $^{Cy3}$R:$^{Cy5}S_{NP}$.

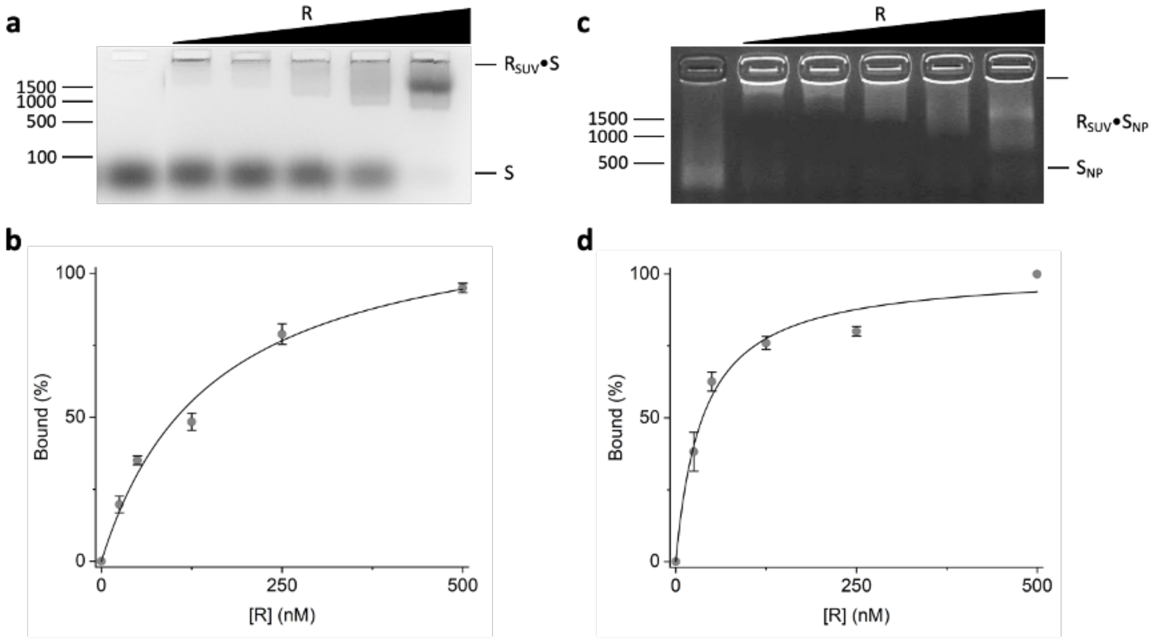


**Figure S18. Gel electrophoretic mobility shift assay to assess the binding of the ssDNA ligand (S) or nanopore-bound ligand ($S_{NP}$) to the receptor (R), which is tethered to the surface of SUVs.** (a) A 3% agarose gel shift assay. Addition of increasing concentrations of R results in the formation of the $R_{SUV}$•S membrane-bound dsDNA complex, which is unable to migrate into the gel matrix and remains in the well. S carries a Cy5 fluorophore to improve resolution at low concentrations. The gel was imaged using a fluorescence gel scanner. (b) Binding curve of $R_{SUV}$•S complexation displayed as percent bound. The data are fit with a Langmuir binding curve and represent averages and standard deviations from 3 independent experiments. (c) A 2% agarose gel shift assay: increasing the concentration of R results in the formation of the $R_{SUV}$•$S_{NP}$ membrane-bound complex, which is unable to migrate into the gel matrix and remains in the well. (d) Binding curve of $R_{SUV}$•$S_{NP}$ complexation displayed as percent bound. The data were fit with a Langmuir binding curve and represent averages and standard deviations from 3 independent experiments. All gels were run in 1x TAE buffer at 60 V for 60 min. The major markers of a 100 bp DNA ladder are indicated on the left.

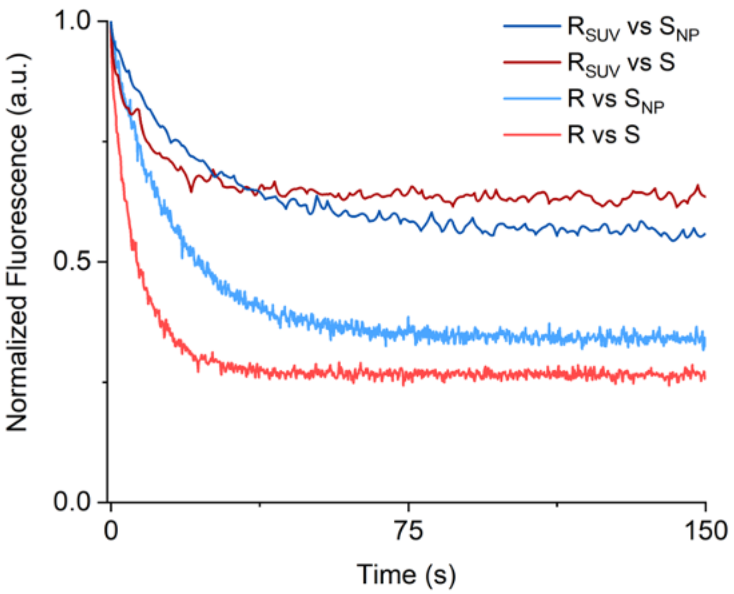


**Figure S19. FRET kinetic analysis of hybridization between R and S (or $S_{NP}$) in solution and on the membrane surface.** The fluorescence signal was normalized as $F/F_0$ from the point of S or $S_{NP}$ addition. For R+S (red) and R+$S_{NP}$ (blue), R was added at 1.25 nM and S and $S_{NP}$ were added in a 100-fold excess at 125 nM. $R_{SUV}$ vs S (dark red) and $R_{SUV}$ vs $S_{NP}$ (dark blue), R was added at 5 to nM and S and $S_{NP}$ were added in a 20-fold excess at 100 nM. SUVs (100 nm) were added at a concentration of 22.2 pM for an R:SUV loading of 225:1 or a lipid:R ratio of 1167:1.

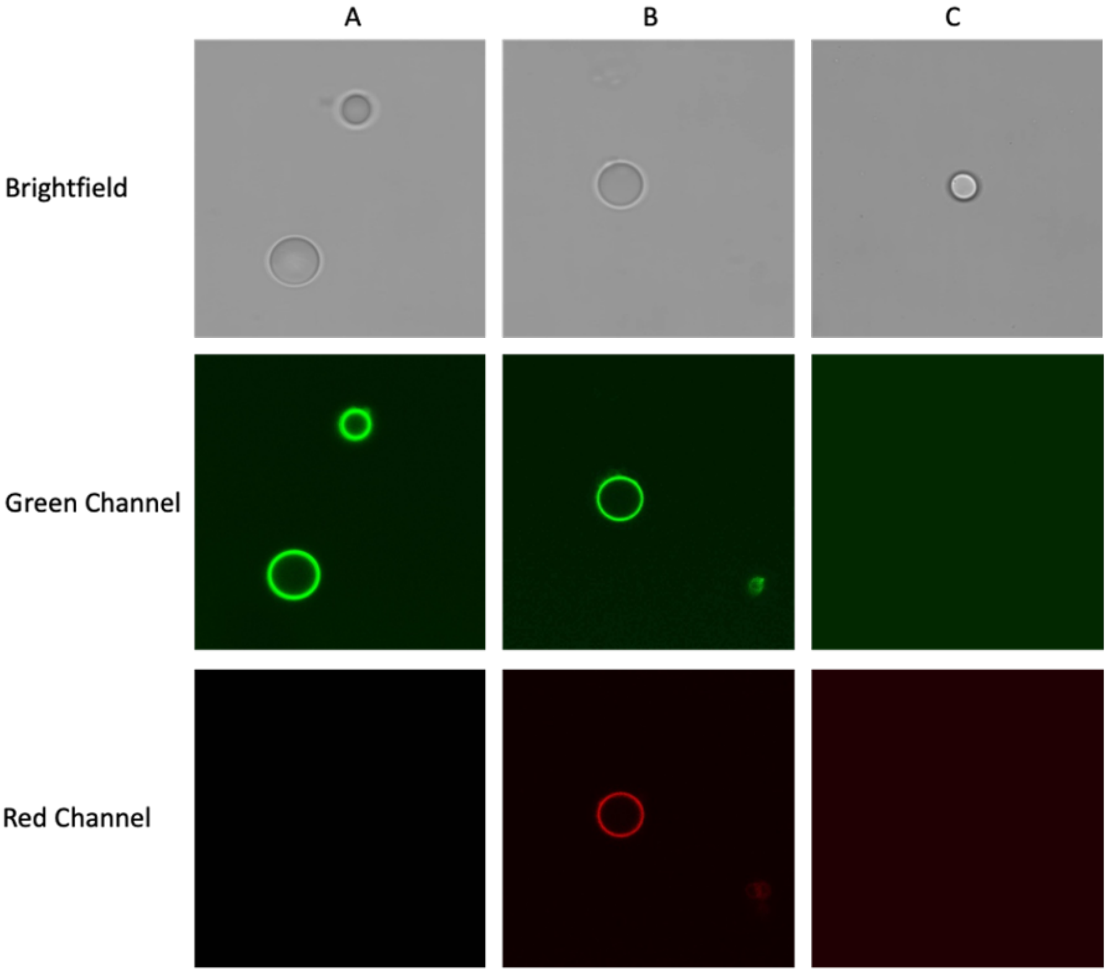


**Figure S20. Confocal microscopy images of hybridization between $S_{NP}$ and R on the surface of GUVs.** Confocal laser scanning microscopy images of the binding of ${}^{Cy3}R_{GUV}$ and ${}^{Cy5}S_{NP}$ on the surface of giant unilamellar vesicles (GUVs) composed of POPC lipids. Three channels are shown: the brightfield in the top row confirms the presence of GUV's, the second row shows the green channel exciting the Cy3 fluorophore on R, the third row shows the red channel exciting the Cy5 on $S_{NP}$. (a) Fluorescent green halos around the GUVs confirm insertion and tethering of ${}^{Cy3}R$ into the bilayer. (b) Both green and red fluorescent halos around the GUVs reaffirm (A) as well as successful hybridisation of Cy5$S_{NP}$ with ${}^{Cy3}R_{GUV}$. (c) The absence of any fluorescent halos confirms that without the presence of Cy3R on the membrane surface Cy5$S_{NP}$ does not interact with the GUVs.

**Table S6: Summary of thermodynamic and kinetic data obtained for the four conditions of the model system.** Assembly in solution for R vs S and R and $S_{NP}$ as well as at the membrane interface when R is bound to SUVs composed of DOPC:DOPE in a 7:3 mole ratio, $R_{SUV}$ vs S; and $R_{SUV}$ vs $S_{NP}$. Averages and standard deviations were obtained from three independent repeats.

| Parameter | Condition | Avg. ± Std. Dev. |
|---|---|---|
| $K_d$ (M) | S vs R | 4.05 ± 0.36 x $10^{-8}$ |
| | $S_{NP}$ vs R | 8.01 ± 0.33 x $10^{-8}$ |
| | S vs $R_{SUV}$ | 15.4 ± 2.36 x $10^{-8}$ |
| | $S_{NP}$ vs $R_{SUV}$ | 7.86 ± 1.91 x $10^{-8}$ |
| $k_{on}$ ($M^{-1}s^{-1}$) | S vs R | 10.7 ± 0.87 x $10^{5}$ |
| | $S_{NP}$ vs R | 4.79 ± 0.24 x $10^{5}$ |
| | S vs $R_{SUV}$ | 9.16 ± 0.89 x $10^{5}$ |
| | $S_{NP}$ vs $R_{SUV}$ | 3.88 ± 0.44 x $10^{5}$ |
| $k_{off}$ ($s^{-1}$) | S vs R | 4.33 ± 0.53 x $10^{-2}$ |
| | $S_{NP}$ vs R | 3.84 ± 0.25 x $10^{-2}$ |
| | S vs $R_{SUV}$ | 14.1 ± 2.60 x $10^{-2}$ |
| | $S_{NP}$ vs $R_{SUV}$ | 3.05 ± 0.82 x $10^{-2}$ |

**Table S7. Summary of FRET efficiencies derived from the hybridization kinetics between the four components of the model system.** Averages and standard deviations were calculated from at least three independent repeats.

| Condition | FRET Efficiency, E<br>Avg. ± Std. Dev. | Extent of reaction<br>(norm. % vs control) |
|---|---|---|
| Control | 0.80 | 100 |
| S vs R | 0.74 ± 0.01 | 92.6 ± 1.77 |
| $S_{NP}$ vs R | 0.66 ± 0.05 | 82.8 ± 8.84 |
| S vs $R_{SUV}$ | 0.37 ± 0.02 | 46.1 ± 3.54 |
| $S_{NP}$ vs $R_{SUV}$ | 0.45 ± 0.06 | 55.8 ± 10.6 |

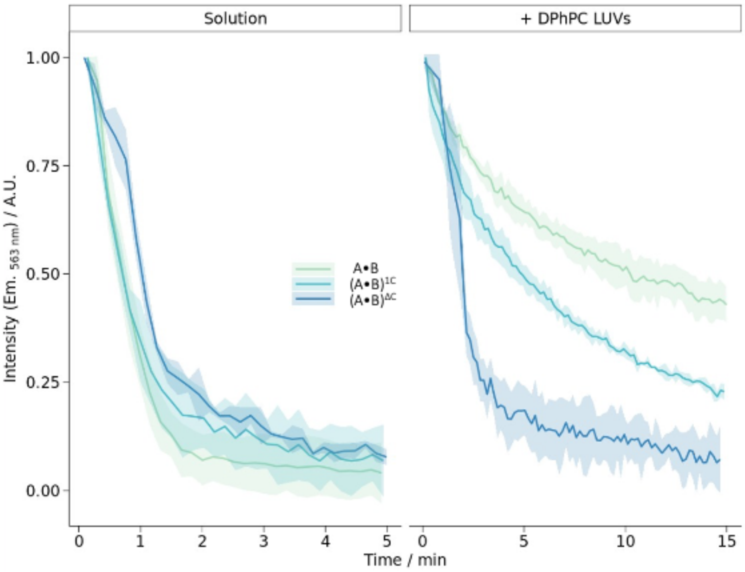


**Figure S21. Digestion assay to confirm pore insertion.** In solution, both $(A\bullet B)^{1C}$ and A•B are fully and rapidly digested by the BAL-31 DNA digestion enzyme. When $(A\bullet B)^{1C}$ and A•B are first incubated with LUVs composed of DPhPC, digestion is slowed in both instances likely because of steric hindrance from the vesicles. Digestion is significantly inhibited by the insertion of A•B into the lipid bilayer making the strand nicks of the DNA inaccessible. In the case of $(A\bullet B)^{1C}$, access to the nicks is reduced by the presence of the membrane, but only on the side with the cholesterol lipid anchor.

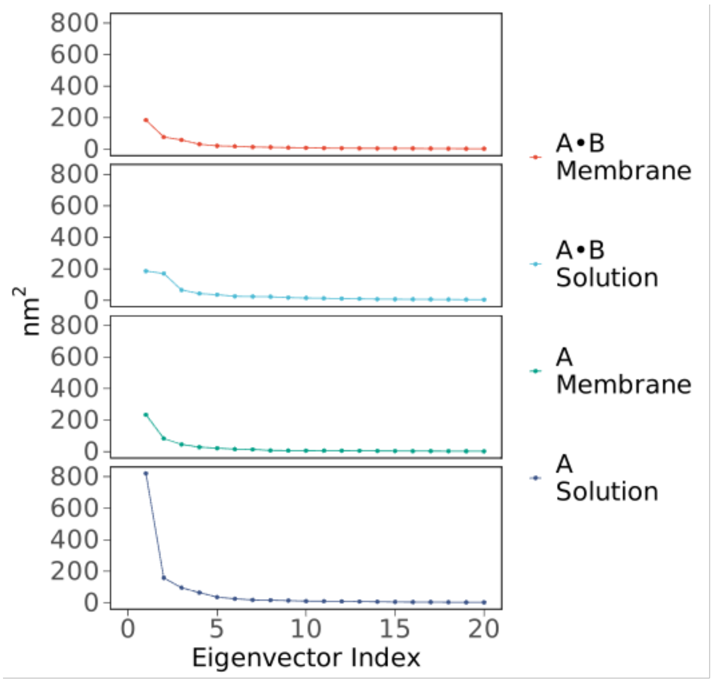


**Figure S22. Eigenvector index showing slowest modes of simulated trajectories.** Eigenvector index was generated through Principle Component Analysis using gmx_covar and gmx_anaeig, demonstrating the first ten slowest modes describe over 90% of the structural dynamics of the DNA heavy atoms.[3]

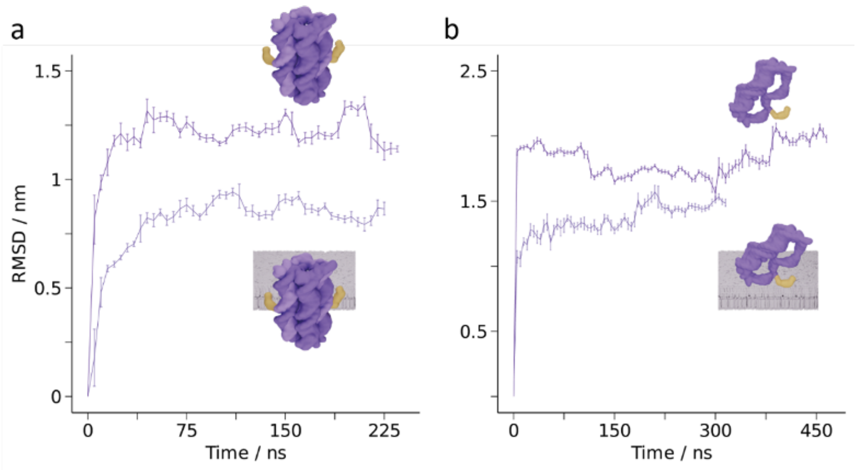


**Figure S23. Time dependent Root Mean Square Deviation (RMSD) of DNA heavy atoms for the 4 simulated trajectories.** (a) RMSD for the nanopore A•B in a 1 M KCl solution (top) or embedded within a POPC bilayer (bottom). (b) RMSD for component A in a 1 M KCl solution (top) or tethered to a POPC bilayer (bottom). The RMSD was calculated using initial idealised structures composed of rigid B-form DNA helices. The data is plotted as a 10 ns running average. Error bars represent the standard deviation.

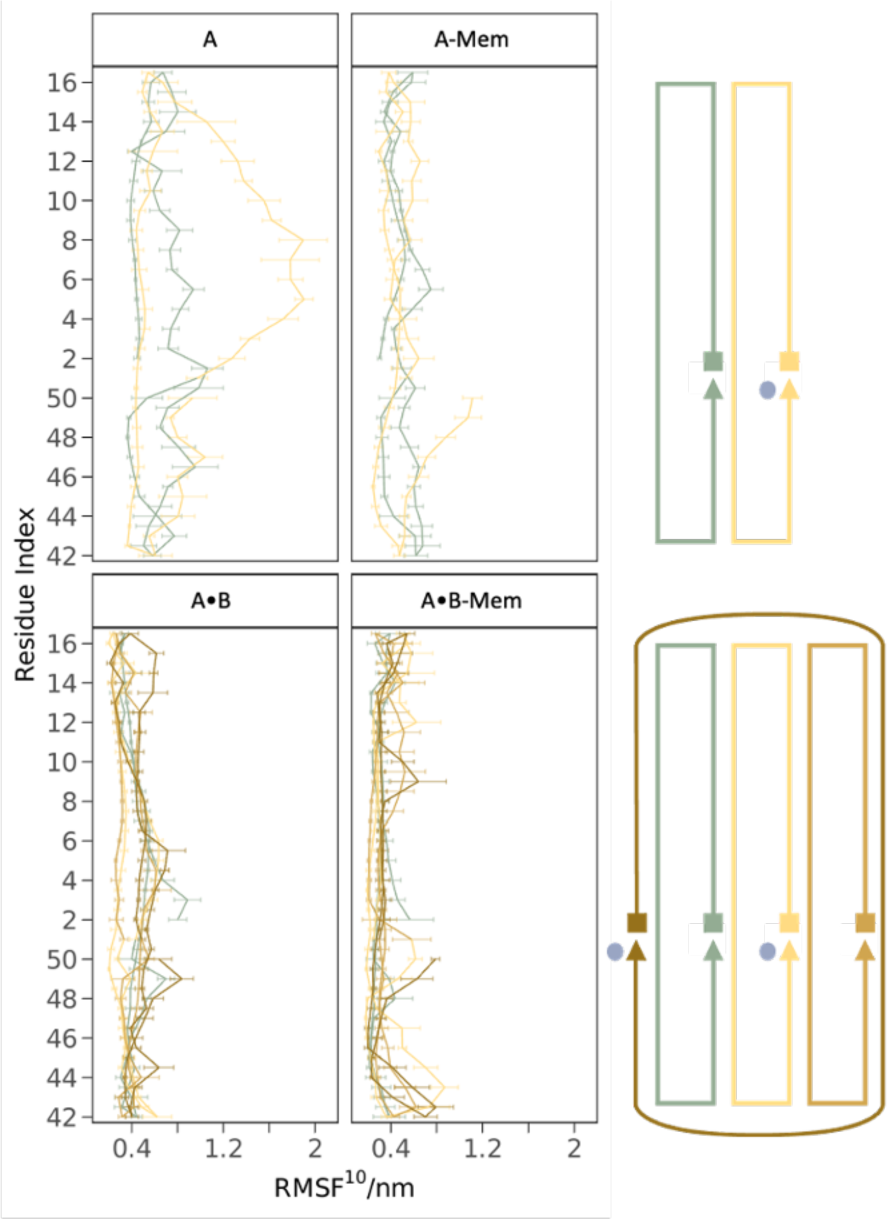


**Figure S24. Per-residue Root Mean Square Fluctuations10 of the DNA nanostructures from the four simulated trajectories.** The residue index on the Y axis has been ordered to match that of the associated strand maps map (Top: A; Bottom: A•B). Squares represent the 5' and triangles the 3' end of each DNA strand. The grey circle indicates a cholesterol modification attached to the 3' end of the DNA strand via tri(ethylene) glycol (TEG) linker.

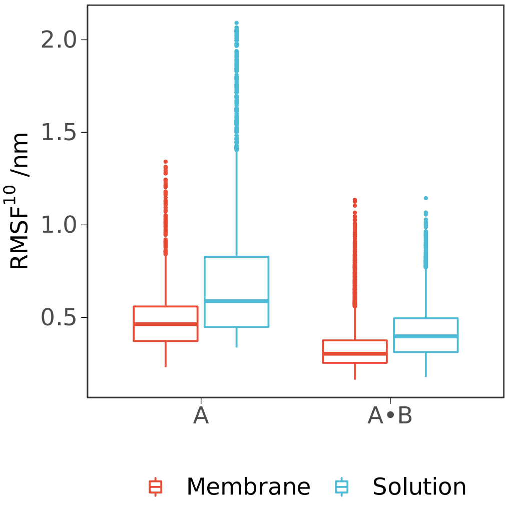


**Figure S25. Boxplot of RMSF$^{10}$ showing the values for Component A and nanopore A•B in solution and interacting with membranes.** The boxplot shows the RMSF$^{10}$ values shown as the arithmetic average of the DNA heavy atoms of each of the four simulated trajectories.

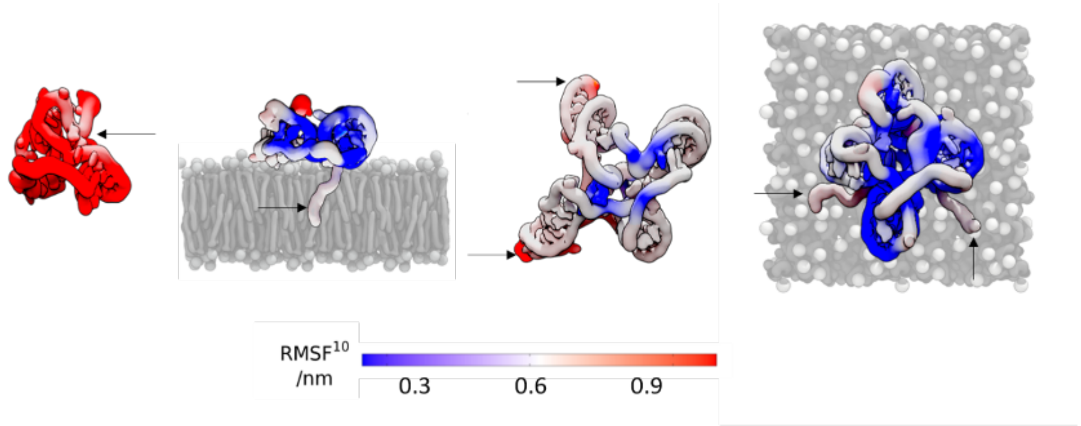


**Figure S26. Top-down images showing representative structures for the four simulated trajectories, coloured by per-residue RMSF$^{10}$.** From left to right; the A subunit in solution, the A subunit tethered to a POPC bilayer, the A•B nanopore in solution and the A•B nanopore embedded in a POPC bilayer. For clarity the solvent has been omitted and the bilayer has been made transparent. The location of the hydrophobic cholesterol anchors has been indicated with a black arrow.

**Table S8. Calculated Cy3-Cy5 distances as an approximation for the average diameter of the nanopore, A•B, in each condition.** Distances were calculated using the FRET efficiencies derived from the pre-folded controls in Table S3 when $R_0$ = 5.6 or 6 nm for Cy3-Cy5.

| Condition | Cy3-Cy5 Distance /nm | |
|---|---|---|
| | $R_0 = 5.6$ | $R_0 = 6.0$ |
| $(A{\bullet}B)^{\Delta C}$ | 6.63 ± 0.47 | 7.10 ± 0.50 |
| $(A{\bullet}B)^{1C}$-SUV | 6.58 ± 0.13 | 7.05 ± 0.14 |
| A•B-SUV | 6.19 ± 0.14 | 6.63 ± 0.15 |

**Table S9. Area per lipid (APL) values for the two membrane simulations.** APLs were calculated using the MEMBPlugin[25] in VMD, and averaged across the NpT production simulations, discarding the initial 10% of the trajectory. The values compare favourably with the literature values for POPC membranes,[30] but show an increased APL for the trajectory with the membrane spanning DNA nanostructure.

| Trajectory | Area Per Lipid ($Å^2$) |
|---|---|
| A•B | 74.49 ± 0.77 |
| A | 71.63 ± 0.07 |

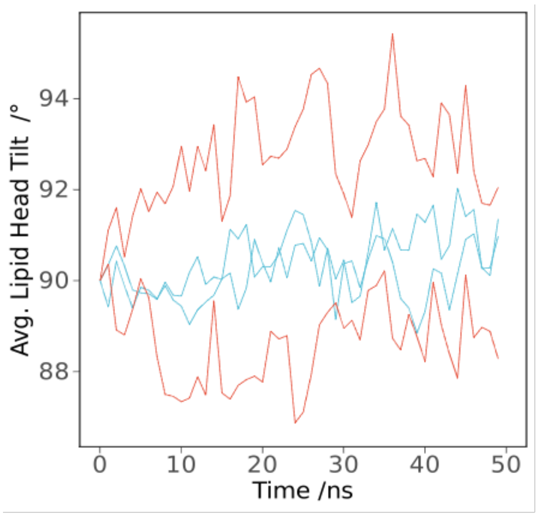


**Figure S27. Average angle of the lipid head groups relative to the bilayer normal.** Insertion of the A•B nanopore results in rapid formation of a toroidal lipid. The headgroups from the trajectory with the membrane spanning A•B DNA nanopore are shown in red and the headgroups from the trajectory with the membrane tethered Component A are shown in blue. The lipids were split by leaflet, and lipids further than 4 nm from the DNA structures were discarded, and the angle calculated with gmx_gangle and time averaged to give an indication of the direction of motion. The initial region of the NvT equilibration trajectory had to be used to capture this motion, as it mostly occurred within the first 20 ns of the simulation.

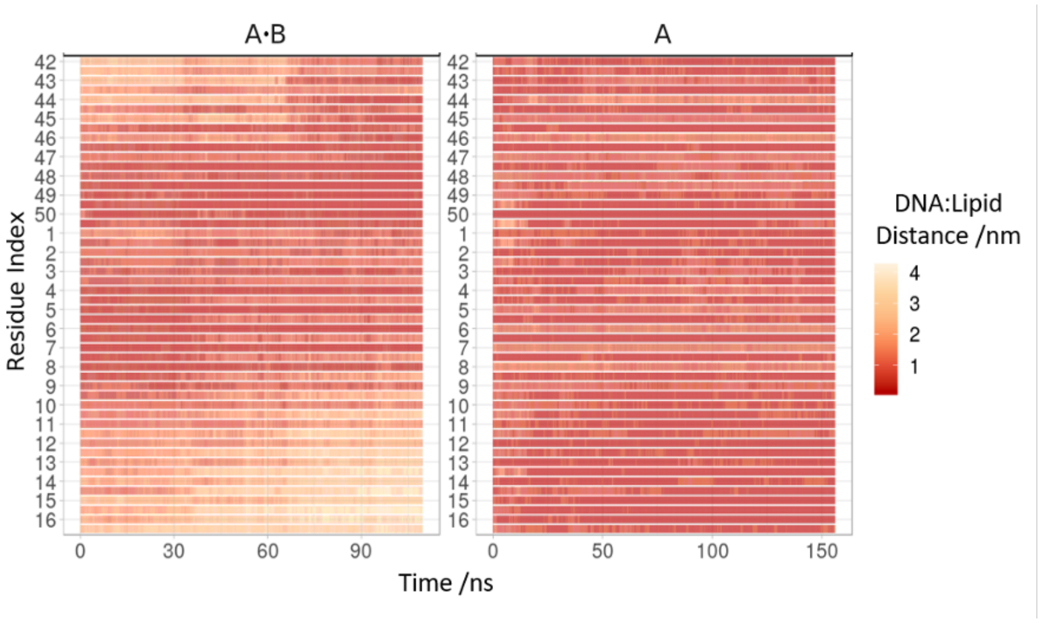


**Figure S28. Evolution of the simulated per residue distance between the lipid bilayer and DNA nanopore, A•B, and component A.** Calculated using *gmx_mindist* over the initial *NvT* equilibration trajectories to demonstrate the DNA nanopore induced lipid remodeling.

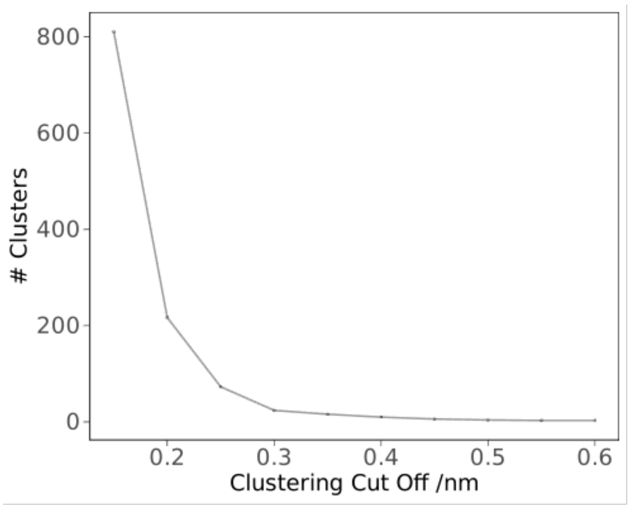


**Figure S29. Analysis of different clustering cut-off values for the clustered lumen analysis of the membrane embedded DNA nanopore, A•B.** Clustering was performed using the gromos method[22] on the NpT region of the trajectory, after discarding the initial 10% of the trajectory. The final clustering cut off used was 0.25 nm, selected to ensure there were no more than 50 clusters, and >10 contained 90% of the trajectory and fewer than 3 clusters containing a single species.

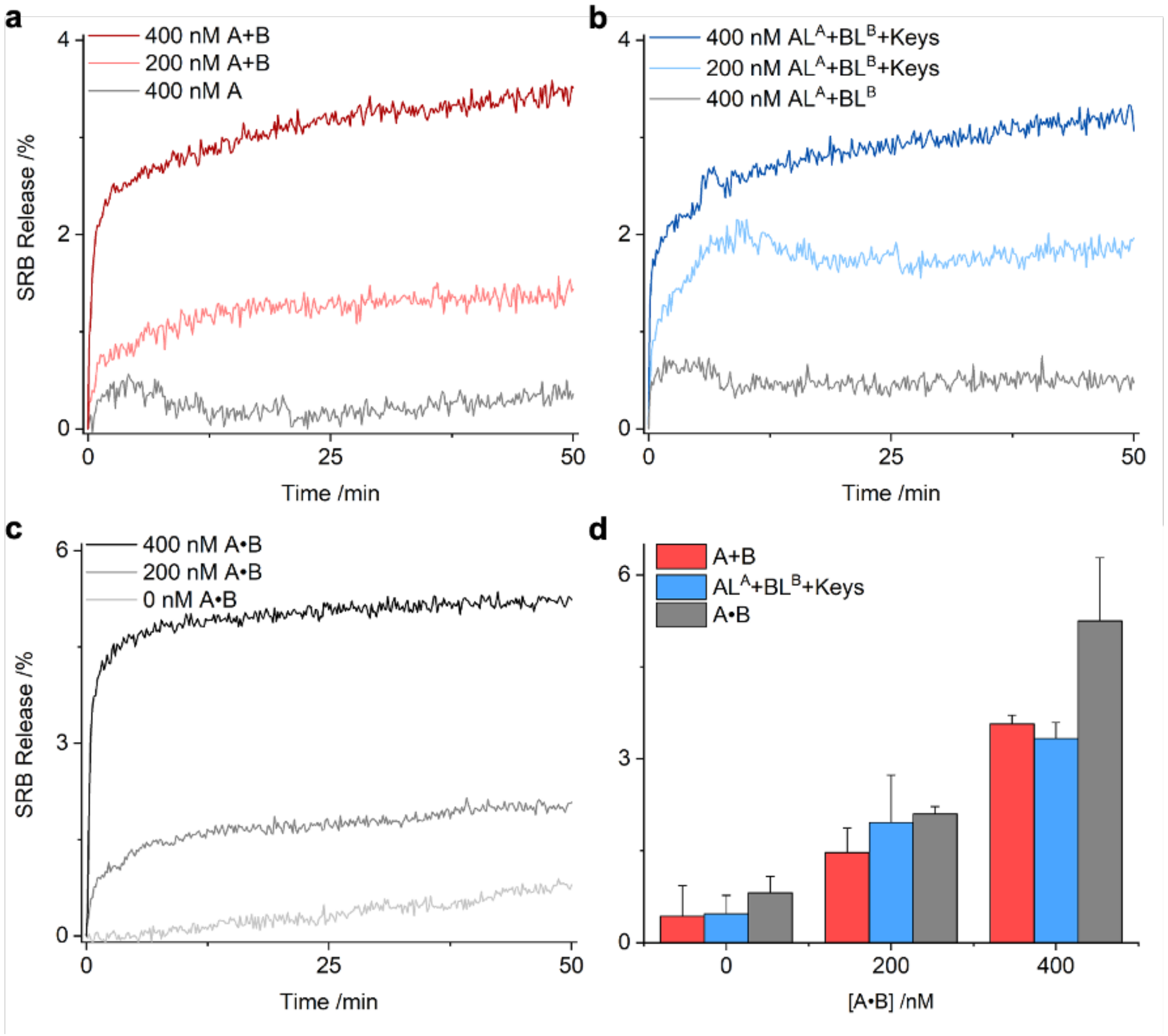


**Figure S30. A•B concentration dependent SRB release from SUVs.** (a) Concentration dependence when A•B was formed from unlocked components A+B (b), from locked components AL$^A$+BL$^B$ upon addition of keys, (c) and pre-folded A•B. (d) Bar chart of net fluorescence increase summarizing the results from (a)-(c). 0 nM A•B represents the controls: 400 nM A only (red), 400 nM (AL$^A$+BL$^B$) (blue) and buffer (grey). The data represent averages and standard deviations from three independent repeats.

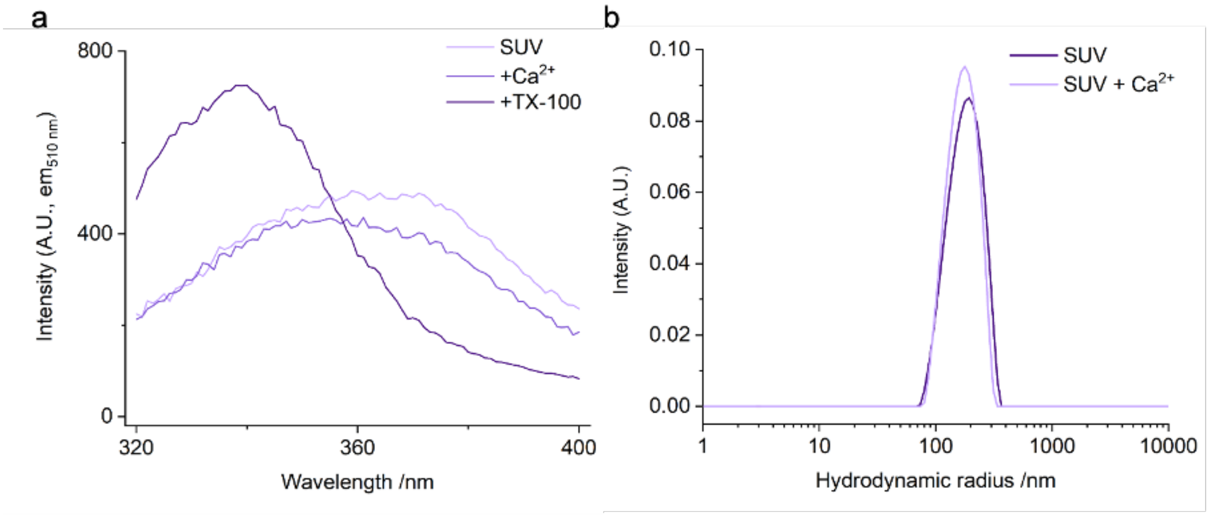


**Figure S31. Characterization of Fura-2-encapsulated vesicles for the $Ca^{2+}$ influx assay.** At low $Ca^{2+}$ concentrations Fura-2 excites at 380 nm. As the concentration of $Ca^{2+}$ increases, the excitation maximum shifts to 340 nm. The emission wavelength remains constant at 510 nm. $Ca^{2+}$ binding can thus be assessed by monitoring the ratio of the excitation intensity at 340/380 nm. (a) Fluorescence scan of purified SUVs with encapsulated Fura-2 (100 μM) demonstrating the absence of $Ca^{2+}$ in the vesicles and a low 340/380 ratio (0.95). Addition of 250 μM $CaCl_2$ results in a mild increase of the 340/380 ratio (1.13) consistent with some unpurified dye and confirms vesicles impermeability to both Fura-2 and $Ca^{2+}$. Upon addition of a detergent (Triton X-100), the vesicles are lysed removing the barrier between Fura-2 and $Ca^{2+}$ and resulting in maximum binding indicated by a significant increase in the 340/380 ratio (5.62). (B) Dynamic light scattering analysis of vesicles formed from POPC with encapsulated Fura-2 (100 μM) in the absence and presence of $Ca^{2+}$. In the absence of $Ca^{2+}$, SUVs had an average diameter of 186 nm, while in the presence of $Ca^{2+}$, SUVs had an average diameter of 178 nm consistent with $Ca^{2+}$ induced membrane compression.

**References**


(1) Magde, D.; Elson, E. L.; Webb, W. W. Fluorescence correlation spectroscopy. Ii. An experimental realization. *Biopolymers* **1974,** *13*, 29-61.

(2) Werner, S.; Ebenhan, J.; Haupt, C.; Bacia, K. A quantitative and reliable calibration standard for dual-color fluorescence cross-correlation spectroscopy. *ChemPhysChem* **2018,** *19*, 3436-3444.

(3) Schwille, P., *Cross-correlation analysis in fcs. In fluorescence correlation spectroscopy*. 1st ed.; Springer: 2001; Vol. 65.

(4) Bacia, K.; Petrasek, Z.; Schwille, P. Correcting for spectral cross-talk in dual-color fluorescence cross-correlation spectroscopy. *ChemPhysChem* **2012,** *13*, 1221-1231.

(5) Jejoong, Y.; N, S. A.; Chen-Yu, L.; Aleksei, A., CaDNAno to pdb file converter. 2014.

(6) Abagyan, R.; Totrov, M.; Kuznetsov, D. Icm-a new method for protein modeling and design: Applications to docking and structure prediction from the distorted native conformation. *J. Comput. Chem.* **1994,** *15*, 488-506.

(7) Vanommeslaeghe, K.; Hatcher, E.; Acharya, C.; Kundu, S.; Zhong, S.; Shim, J.; Darian, E.; Guvench, O.; Lopes, P.; Vorobyov, I.; Mackerell, A. D. Charmm general force field: A force field for drug-like molecules compatible with the charmm all-atom additive biological force fields. *J. Comput. Chem.* **2009,** *31*, 671-690.

(8) DeLano, W. L., Pymol | pymol.Org. 2002.

(9) Ao, J.; Ribeiro, V.; Radak, B.; Stone, J.; Gullingsrud, J.; Saam, J.; Phillips, J. *Psfgen user's guide*; 2020.

(10) Yoo, J.; Li, C.-Y.; Slone, S. M.; Maffeo, C.; Aksimentiev, A., A practical guide to molecular dynamics simulations of DNA origami systems. Humana Press Inc.: 2018; Vol. 1811, pp 209-229.

(11) Maffeo, C.; Bhattacharya, S.; Yoo, J.; Wells, D.; Aksimentiev, A. Modeling and simulation of ion channels. *Chem. Rev.* **2012,** *112*, 6250-6284.

(12) Humphrey, W.; Dalke, A.; Schulten, K. Vmd: Visual molecular dynamics. *J. Mol. Graph. Model.* **1996,** *14*, 33-38.

(13) Kessel, A.; Ben-Tal, N.; May, S. Interactions of cholesterol with lipid bilayers: The preferred configuration and fluctuations. *Biophys. J.* **2001,** *81*, 643-658.

(14) Aksimentiev, A.; Sotomayor, M.; Wells, D.; Huang, Z. Membrane proteins tutorial. *Theoretical and Computational Biophysics Group, University of Illinois at Urbana-Champaign* **2009**.

(15) Davidchack, R. L.; Handel, R.; Tretyakov, M. V. Langevin thermostat for rigid body dynamics. *J. Chem. Phys.* **2009,** *130*, 234101-234101.

(16) Feller, S. E.; Zhang, Y.; Pastor, R. W.; Brooks, B. R. Constant pressure molecular dynamics simulation: The langevin piston method. *J. Chem. Phys.* **1995,** *103*, 4613-4621.

(17) Phillips, J. C.; Braun, R.; Wang, W.; Gumbart, J.; Tajkhorshid, E.; Villa, E.; Chipot, C.; Skeel, R. D.; Kale, L.; Schulten, K. Scalable molecular dynamics with namd. *J. Comput. Chem.* **2005,** *26*, 1781-1802.

(18) Essmann, U.; Perera, L.; Berkowitz, M. L.; Darden, T.; Lee, H.; Pedersen, L. G. A smooth particle mesh ewald method. *J. Chem. Phys.* **1995,** *103*, 8577-8593.

(19) Miyamoto, S.; Kollman, P. A. Settle - an analytical version of the shake and rattle algorithm for rigid water models. *J. Comput. Chem.* **1992,** *13*, 952-962.

(20) Darden, T.; York, D.; Pedersen, L. Particle mesh ewald: An n ·log( n ) method for ewald sums in large systems. *J. Chem. Phys.* **1993,** *98*, 10089-10092.

(21) Makov, G.; Payne, M. C. Periodic boundary conditions in ab initio calculations. *Phys. Rev. B* **1995,** *51*, 4014-4022.

(22) Van Der Spoel, D.; Lindahl, E.; Hess, B.; Groenhof, G.; Mark, A. E.; Berendsen, H. J. C. Gromacs: Fast, flexible, and free. *J. Comput. Chem.* **2005,** *26*, 1701-1718.

(23) Ginestet, C. Ggplot2: Elegant graphics for data analysis. *J. Royal Stat. Society-Series A* **2011,** *174*, 245-246.

(24) Racine, J. S. Rstudio: A platform-independent ide for r and sweave. *J. Appl. Econ.* **2012,** *27*, 167-172.

(25) Ramanathan, A.; Agarwal, P. K. Computational identification of slow conformational fluctuations in proteins. *J. Phys. Chem. B* **2009,** *113*, 16669-16680.

(26) Daura, X.; Gademann, K.; Jaun, B.; Seebach, D.; van Gunsteren, W. F.; Mark, A. E. Peptide folding: When simulation meets experiment. *Ang. Chem. Int. Ed.* **1999,** *38*, 236-240.

(27) Smart, O. S.; Neduvelil, J. G.; Wang, X.; Wallace, B. A.; Sansom, M. S. P. Hole: A program for the analysis of the pore dimensions of ion channel structural models. *J. Mol. Graph.* **1996,** *14*, 354-360.

(28) Giorgino, T. Computing 1-d atomic densities in macromolecular simulations: The density profile tool for vmd. *Comput. Phys. Commun.* **2014,** *185*, 317-322.

(29) Guixà-González, R.; Rodriguez-Espigares, I.; Ramírez-Anguita, J. M.; Carrió-Gaspar, P.; Martinez-Seara, H.; Giorgino, T.; Selent, J. Membplugin: Studying membrane complexity in vmd. *Bioinformatics* **2014,** *30*, 1478-1480.

(30) Lantzsch, G.; Binder, H.; Heerklotz, H. Surface area per molecule in lipid/c12e n membranes as seen by fluorescence resonance energy transfer. *J. Fluoresc.* **1994,** *4*, 339-343.
